# Supplementary material for: Loss-of-function of an α-SNAP gene confers resistance to soybean cyst nematode
Source: Nat Commun. 2023 Nov 22;14:7629. doi: 10.1038/s41467-023-43295-y (PMC10665432; doi:10.1038/s41467-023-43295-y)
Supplement: Supplementary file 1 — Supplementary Information [file 41467_2023_43295_MOESM1_ESM.pdf]

QTL MAPPING
SUPPLEMENTARY DATA

Supplementary Figure 1. Descriptive statistics of responses to SCN TN22 population of HG type 1.2.5.7 of three populations: PI 90763 x Peking, Forrest x PI 437654, and SA10-8471 x PI 90763. The parental lines were added to evaluate resistance differences. The normality tests of female index (%) are shown by Shapiro-Wilk (*w*), skewness, and kurtosis.

Female Index [%]						Shapiro-Wilk (*w*)	Skewness	Kurtosis
**Parents**		**144 $F_{3:4}$**						
PI 90763	Peking	Mean	Min	Max	SD			
0	19.8	7.3	0	24.1	6.2	0.92	0.54	2.4

Female Index [%]						Shapiro-Wilk (*w*)	Skewness	Kurtosis
**Parents**		**131 $F_{3:4}$**						
Forrest	PI 437654	Mean	Min	Max	SD			
86	0.6	45.7	0	107	35.3	0.62	0.02	-1.7

Female Index [%]						Shapiro-Wilk (*w*)	Skewness	Kurtosis
**Parents**		**244 $F_{3:4}$**						
SA10-8471	PI 90763	Mean	Min	Max	SD			
88	0	74.3	0	114	28.1	0.83	-1.4	1.0

Supplementary Figure 2. (a) Genetic linkage maps created for 144 $F_{3:4}$ lines from population PI 90763 x Peking. The X-axis represents chromosome numbers, and Y-axis represents genetic position of single nucleotide polymorphism (SNP) markers. Distribution of SNPs are represented by black bars across each chromosome.

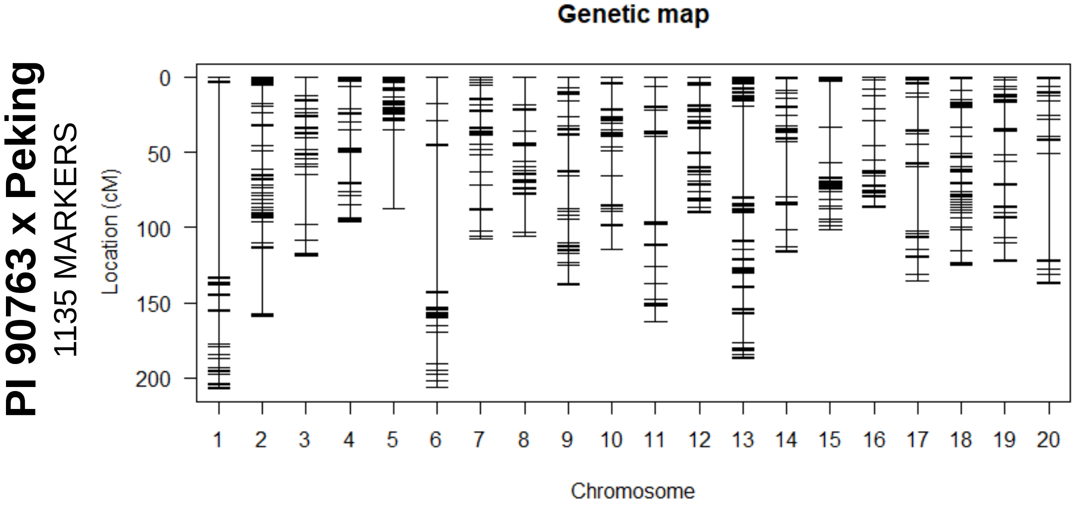

Chr #	Markers assigned to parents	Markers removed for distortion, uninformative	Markers removed for gap closure	Final number of markers	Final Length (cM)	Average spacing (cM)	Max spacing (cM)
1	56	205	1	55	213.7	0.3	128.4
2	83	239	-	83	158.9	0.3	43.9
3	56	219	-	56	119.0	0.3	32.8
4	44	228	-	44	96.4	0.6	19.7
5	41	252	1	40	87.2	0.6	52.3
6	64	250	-	64	206.5	0.0	97.0
7	50	269	-	50	107.8	0.3	15.8
8	49	333	1	48	144.8	0.3	24.8
9	58	207	-	58	138.5	0.6	23.5
10	40	277	-	40	114.9	0.6	19.5
11	37	235	1	36	187.7	0.5	56.9
12	50	209	1	49	90.3	0.8	15.5
13	115	271	-	115	186.7	0.3	60.3
14	37	236	-	37	116.0	0.6	36.7
15	83	220	-	83	101.3	0.0	29.9
16	32	207	-	32	86.3	0.8	16.5
17	40	221	-	40	135.4	0.6	42.8
18	80	298	-	80	125.1	0.3	13.3
19	90	237	-	90	122.5	0.0	17.2
20	35	237	-	35	137.5	0.4	70.5
Overall	1140	4850	5	1135	2676.3	0.4	128.4

Supplementary Figure 2. (b) Genetic linkage maps created for 131 $F_{3:4}$ lines from population Forrest x PI 437654. The X-axis represents chromosome numbers, and Y-axis represents genetic position of single nucleotide polymorphism (SNP) markers. Distribution of SNPs are represented by black bars across each chromosome.

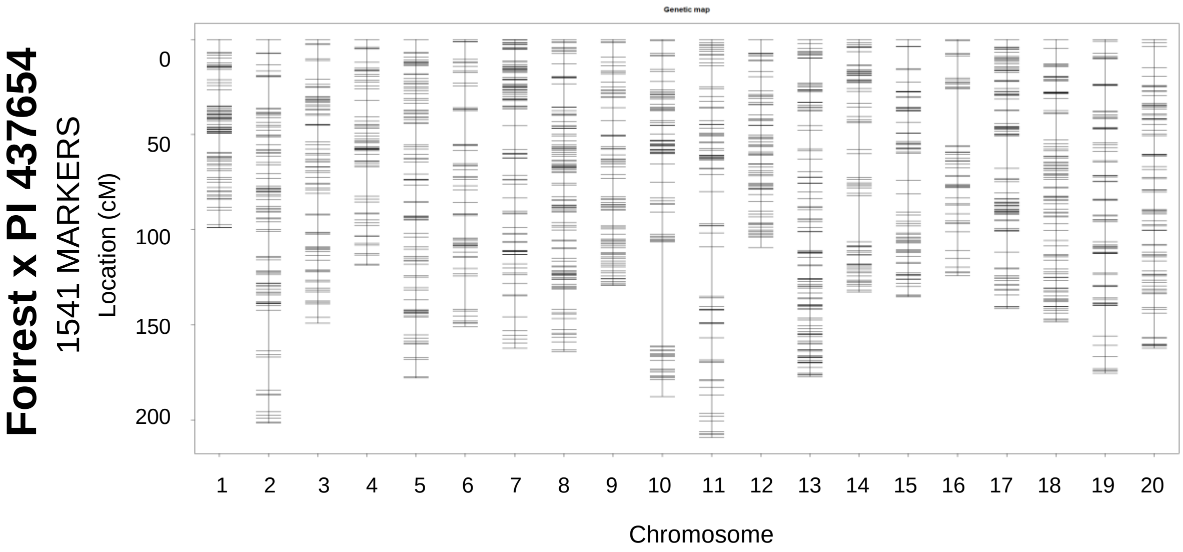

Chr #	Markers assigned to parents	Markers removed for distortion, uninformative	Markers removed for gap closure	Final number of markers	Final Length (cM)	Average spacing (cM)	Max spacing (cM)
1	134	61	-	73	99.1	1.4	9.9
2	144	52	2	90	201.7	2.3	21.4
3	135	60	-	75	149.1	2.0	11.0
4	134	76	1	57	118.7	2.1	15.4
5	149	61	-	88	177.9	2.0	11.3
6	124	64	1	59	150.8	2.6	17.7
7	172	82	-	90	162.4	1.8	12.3
8	183	80	-	103	164.1	1.6	12.3
9	110	36	-	74	129.3	1.8	9.6
10	162	85	2	75	187.8	2.5	54.8
11	120	42	3	75	209.3	2.8	26.2
12	115	47	-	68	109.2	1.6	7.4
13	182	78	-	104	177.2	1.7	13.1
14	103	36	-	67	132.8	2.0	24.4
15	130	62	-	68	135.3	2.0	14.2
16	106	60	-	46	124.1	2.8	29.5
17	154	66	-	88	141.6	1.6	16.8
18	167	80	-	87	148.4	1.7	14.0
19	169	94	-	75	175.5	2.4	16.2
20	121	42	-	79	162.4	2.1	12.6
Overall	2814	1264	9	1541	3056.8	2.0	54.8

Supplementary Figure 2. (c) Genetic linkage maps created for 244 $F_{3:4}$ lines from population SA10-8471 x PI 90763. The X-axis represents chromosome numbers, and Y-axis represents genetic position of single nucleotide polymorphism (SNP) markers. Distribution of SNPs are represented by black bars across each chromosome.

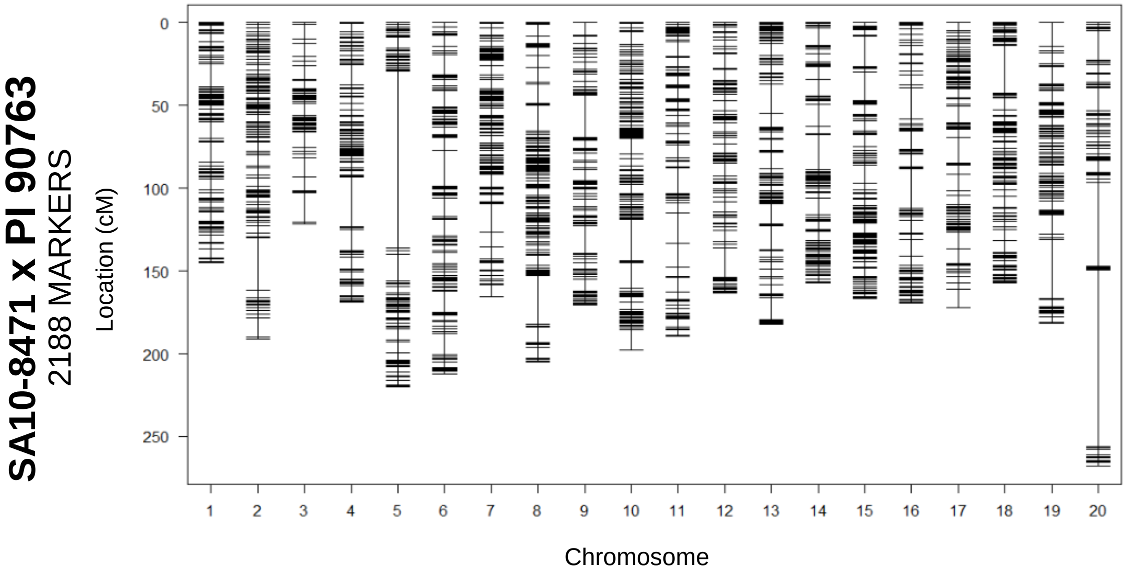

Chr #	Markers assigned to parents	Markers removed for distortion, uninformative	Markers removed for gap closure	Final number of markers	Final Length (cM)	Average spacing (cM)	Max spacing (cM)
1	105	156	1	104	150.0	1.4	14.2
2	131	191	1	130	196.0	1.5	31.6
3	72	203	-	65	200.2	1.9	17.7
4	111	161	-	111	168.6	1.5	30.1
5	92	201	3	89	225.3	2.5	106.7
6	124	190	-	124	212.1	1.7	21.7
7	130	189	-	130	165.4	1.3	17.1
8	144	238	-	144	205.0	1.4	29.6
9	96	169	-	96	170.5	1.8	25.6
10	148	169	-	148	197.9	1.3	24.8
11	96	173	-	91	189.6	2.1	18.5
12	100	159	-	100	168.2	1.6	18.2
13	102	284	2	100	202.4	1.8	17.9
14	100	173	-	100	162.2	1.6	20.8
15	126	177	-	126	166.8	1.3	18.2
16	94	145	-	94	169.3	1.8	24.2
17	113	148	-	113	172.2	1.5	15.3
18	128	250	-	128	162.2	1.2	28.9
19	114	213	-	114	181.7	1.6	35.9
20	81	191	-	81	268.0	3.3	106.9
Overall	2207	3780	7	2188	3733.3	1.7	106.9

Supplementary Figure 3. Quantitative trait loci (QTL) for resistance to SCN TN22 population of HG type 1.2.5.7 mapped in 144 $F_{3:4}$ lines of PI 90763 x Peking, 131 $F_{3:4}$ lines of Forrest x PI 437654, and 244 $F_{3:4}$ lines of SA10-8471 x PI 90763. The analysis was done using MapQTL version 6 software.

Population	Peak Marker [a]	Confidence Interval Markers [b]	Confidence Interval	LOD	PV [c]	Add
PI 90763 x Peking	Gm02_46462690	Gm02_42012522 – Gm02_46907259	4.9 Mb	8.9	22.9	-5.4
	Gm12_9059057	Gm12_7791511 – Gm12_9149774	1.4 Mb	4.1	9.6	-2.3
Forrest x PI 437654	Gm02_45106877	Gm02_43556059 – Gm02_45106877	1.6 Mb	4.4	3.1	8.6
	Gm11_32959788	Gm11_32276359 – Gm11_33309696	1.1 Mb	29.1	35.9	28.6
SA10-8471 x PI 90763	Gm11_32959788	Gm11_32586847 – SNAP11-1	381 Kb	22.3	26.9	17.2
	Gm18_1562536	Gm18_1427672 – SNAP18-1	217 Kb	15.9	18.2	14.1

[a] Peak and confidence interval physical position based on Wm82.a2.v1 are represented by marker names.
[b] SNAP11-1 (Gm11_32968127) and SNAP18-1 (Gm18_1645012) based on Wm82.a2.v1.
[c] Percentage of phenotypic variation represented by individual QTL.

Supplementary Figure 4. Quantitative trait loci (QTL) for resistance to SCN TN22 population of HG type 1.2.5.7 mapped in 144 $F_{3:4}$ lines of PI 90763 x Peking, 131 $F_{3:4}$ lines of Forrest x PI 437654, and 244 $F_{3:4}$ lines of SA10-8471 x PI 90763. Analysis was done using RQTL: (a) Composite Interval Mapping (CIM). Genome wide significance threshold (LOD) indicated by horizontal bars. (b) Output of fitqtl ANOVA using QTL detected by CIM.

a

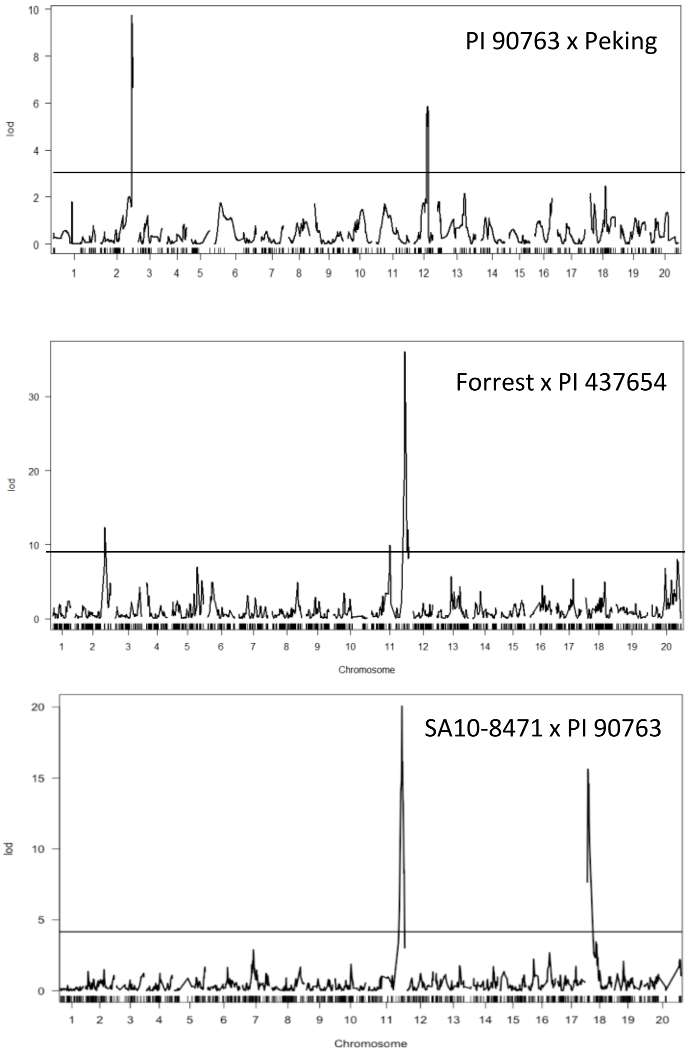

b

Population	Chr.	Peak Marker (W82.a2)	QTL Peak (cM)	1.5 LOD Drop (cM)	LOD Score	LOD Threshold
PI 90763 x Peking	2	Gm02_46462690	153	113.3 - 158.4	9.8	3
	12	Gm12_11867391	66	59.5 - 70.8	5.9	3
Forrest x PI 437654	2	Gm02_45106877	185	165.72-184.58	12.3	9.3
	11	Gm11_32959788	187	182.8-196.7	36	9.3
SA10-8471 x PI 90763	11	Gm11_32889668	319	316.9 – 322.3	76.6	4.3
	18	SNAP18-1	18	14.4 – 18.8	71.3	4.3

Supplementary Figure 5. Estimated allelic effects of all detected QTL in three populations through modeling using R/QTL: (a) Output of fitqtl ANOVA function using QTL detected by Compositive Interval Mapping; (b) Plot of the estimated allelic effects ±1 SE evaluating the combination of two QTL detected by Compositive Interval Mapping.

a

Population	QTL	df	Type III SS	LOD	% Var	F value	P value (Chi^2)	P value (F)	Significance
PI 90763 x Peking	QTL02	6	1,289	9.4	23.6	7.9	0	2.82E-07	***
	QTL12	6	882	6.7	16.1	5.4	0	5.21E-05	***
	QTL02 x QTL12	4	259	2.1	4.7	2.4	0.044	0.0553	
Forrest x PI 437654	QTL02	6	12588	11.556	7.727	10.191	0	4.01E-09	***
	QTL11	6	125846	51.018	77.248	101.879	0	< 2e-16	***
	QTL02 x QTL11	4	4391	4.584	2.696	5.333	0	0.000543	***
SA10-8471 x PI 90763	QTL11	6	112,455	76.6	57.9	128.1	0	< 2E-16	***
	QTL18	6	98,445	71.3	50.7	112.1	0	< 2E-16	***
	QTL11 x QTL18	4	60,160	53.4	30.9	102.8	0	< 2E-16	***

b

PI 90763 x Peking

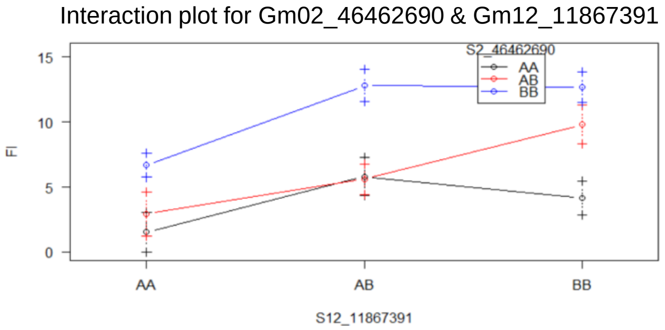

Forrest x PI 437654

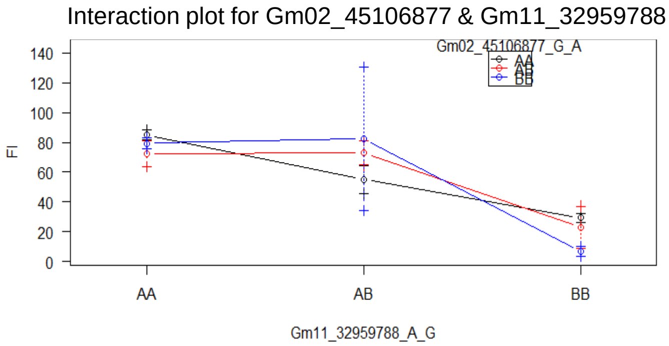

SA10-8471 x PI 90763

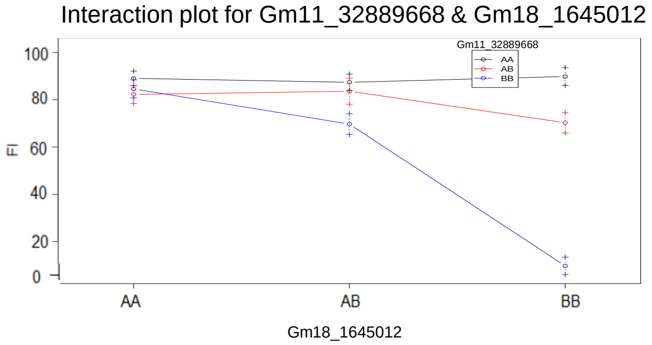

FINE-MAPPING QTL02 SUPPLEMENTARY DATA

Supplementary Figure 6. Initial narrowing-down the region of QTL02 using $F_{3:4}$ recombinant lines derived from populations (a) PI 90763 x Peking; (b) SA10-8471 x PI 90763; and populations reported by Basnet et al. 2022: (c) SA13-1385 x PI 90763, and (d) LD11-2170 x PI 90763. All lines carry homozygous *rhg1-a*, *rhg2*, and *Rhg4*. All lines were inoculated with the same population TN22 of HG type 1.2.5.7. PI 90763 sequence marked in dark gray, the other parent sequence marked in white, and heterozygotes are marked in purple. Red vertical bars indicate recombination spots.

a

F3:4 LINE NAME	Pedigree	*rhg1-a*	*rhg2*	*Rhg4*	Gm02_42012522	Gm02_46462690	Gm02_46537888	Gm02_46907259	FI
19AS-84-2-96	PI 90763 X Peking	YES	YES	YES	CC	TT	AA	GG	0.0
19AS-84-2-102	PI 90763 X Peking	YES	YES	YES	CC	TT	AA	GG	0.0
19AS-84-1-8	PI 90763 X Peking	YES	YES	YES	TT	CC	CC	AA	0.2
19AS-84-2-103	PI 90763 X Peking	YES	YES	YES	TT	CC	CC	AA	0.4
19AS-84-1-19	PI 90763 X Peking	YES	YES	YES	CC	TT	AA	GG	7.1
19AS-84-3-39	PI 90763 X Peking	YES	YES	YES	CC	TT	AA	GG	7.7
19AS-84-2-105	PI 90763 X Peking	YES	YES	YES	CC	TT	AA	GG	9.4
19AS-84-2-110	PI 90763 X Peking	YES	YES	YES	CC	TT	AA	GG	15.8
19AS-84-4-60	PI 90763 X Peking	YES	YES	YES	TT	CC	CC	AA	13.8
19AS-84-2-112	PI 90763 X Peking	YES	YES	YES	TT	CC	CC	AA	9.1

Gm02: 42012522-46462690 (4.45 Mbp)

b

F3:4 LINE NAME	Pedigree	*rhg1-a*	*rhg2*	*Rhg4*	Gm02_42012522	Gm02_43112945	Gm02_44180381	Gm02_44702951	Gm02_45106877	Gm02_45303319	Gm02_45505123	Gm02_45516123	Gm02_45743148	Gm02_45795780	Gm02_45991978	Gm02_46049292	Gm02_46310608	Gm02_46697848	Gm02_46907259	FI
19AS-83-17	SA10-8471 x PI 90763	YES	YES	YES	TT	CC	CC	CC	AA	GG	CC	TT	CC	AA	TT	CC	TT	AA	GG	0.3
19AS-83-79	SA10-8471 x PI 90763	YES	YES	YES	TT	CC	CC	TT	GG	AA	AA	CC	TT	GG	CC	TT	GG	GG	AA	11.4
19AS-83-242	SA10-8471 x PI 90763	YES	YES	YES	CC	TT	TT	TT	GG	AA	AA	CC	TT	GG	CC	TT	GG	GG	AA	11.1
19AS-83-249	SA10-8471 x PI 90763	YES	YES	YES	CC	TT	TT	TT	GG	AA	AA	CC	TT	GG	CC	TT	GG	GG	AA	17.7
19AS-83-260	SA10-8471 x PI 90763	YES	YES	YES	CC	TT	TT	TT	GG	AA	AA	CC	TT	GG	CC	TT	GG	GG	AA	16.2

Gm02: 44180381-46907259 (2.73 Mbp)

c

F3:4 LINE NAME	Pedigree	*rhg1-a*	*rhg2*	*Rhg4*	Gm02_41975550	Gm02_42088045	Gm02_42215041	Gm02_42291659	Gm02_42370864	Gm02_43976713	Gm02_44037267	Gm02_44226448	Gm02_44306758	Gm02_44427664	Gm02_44702951	Gm02_45106877	Gm02_45303319	Gm02_45743148	Gm02_45991978	Gm02_46049292	Gm02_46310608	Gm02_46697848	Gm02_46907259	FI
SA18-17421	SA13-1385 x PI 90763	YES	YES	NO	GG	GG	AA	AA	TT	AA	TT	TT	AA	GG	CC	AA	GG	CC	TT	CC	TT	GG	AA	0.2
SA18-17187	SA13-1385 x PI 90763	YES	YES	HET	GG	GG	AA	AA	TT	GG	GG	CC	GG	AA	TT	GG	AA	TT	CC	TT	GG	AA	GG	8.1
SA18-17499	SA13-1385 x PI 90763	YES	YES	NO	GG	GG	AA	AA	TT	GG	GG	CC	GG	AA	TT	AA	GG	CC	TT	CC	TT	AA	GG	6.7

Gm02: 42370864-45106877 (2.74 Mbp)

d

F3:4 LINE NAME	Pedigree	*rhg1-a*	*rhg2*	*Rhg4*	Gm02_41975550	Gm02_42088045	Gm02_44037267	Gm02_44226448	Gm02_44441196	Gm02_44702951	Gm02_45106877	Gm02_45743148	Gm02_45795780	Gm02_45991978	Gm02_46049292	Gm02_46697848	Gm02_46907259	FI
SA18-18341	LD11-2170 x PI 90763	YES	YES	YES	AA	AA	AA	TT	CC	CC	AA	CC	AA	TT	CC	AA	GG	0.3
SA18-18313	LD11-2170 x PI 90763	YES	YES	HET	AA	AA	AA	TT	CC	CC	AA	CC	AA	TT	CC	GG	AA	0.4
SA18-18445	LD11-2170 x PI 90763	YES	YES	HET	GG	GG	GG	CC	TT	TT	GG	TT	GG	CC	TT	GG	AA	13.2
SA18-18327	LD11-2170 x PI 90763	YES	YES	YES	GG	GG	GG	CC	TT	TT	GG	TT	GG	CC	TT	AA	GG	12.6
SA18-18401	LD11-2170 x PI 90763	YES	YES	NO	AA	AA	GG	CC	TT	TT	GG	CC	AA	TT	CC	AA	AG	14.7
SA18-18102	LD11-2170 x PI 90763	YES	YES	HET	AA	AA	GG	CC	TT	TT	AA	CC	AA	TT	CC	AA	GG	19.2
SA18-18165	LD11-2170 x PI 90763	YES	YES	HET	GG	GG	AA	TT	TT	TT	GG	TT	GG	CC	TT	GG	AA	23.6

Gm02: 44226448-45106877 (0.88 Mbp)

Supplementary Figure 7. Competitive Allele Specific PCR (KASP) assays developed for fine-mapping of QTL02. The QTL02 has been fine-mapped between markers Gm02-09 and Gm02-14.

ASSAY	Wm82	PEKING	PI 90763	POSITION (Wm82.a2)	Primer-Forward_AlleleX	Primer-Forward_AlleleY	Primer-Reverse_Common
MU-Gm02-01	T	A	T	Gm02:44231725	ACAACTGCCACAGCTTTGAAAGCA	ACAACTGCCACAGCTTTGAAAGCT	CAGCAAACACCTTTTTTCTTTCAATGTGAT
MU-Gm02-02	G	T	G	Gm02:44271063	AAATCCAACCACTAGAACCCACACA	TCCAACCACTAGAACCCACACC	TGGACAACAATGTATTTGGTGAATGCATTT
MU-Gm02-03	T	C	T	Gm02:44492572	CCGACAAGATTTGGCAATGTCAG	GCCGACAAGATTTGGCAATGTCAA	CCCCTATATTTGCATTCACGAGCCAA
MU-Gm02-04	C	T	C	Gm02:44553129	GAGCCGTGAAGAAATTGTTGGTGATA	AGCCGTGAAGAAATTGTTGGTGATG	TGTAATCCATTTCTTCTACACCATCAGCA
MU-Gm02-05	A	G	A	Gm02:44583850	TATTTAAGAGTTAAAGTATTTTTGAATGTATTC	TTATTTAAGAGTTAAAGTATTTTTGAATGTATTT	CTCCAGTACTTGATATGATGTCTGTTTAAA
MU-Gm02-07	G	A	G	Gm02:44600617	TCCAAAATTTGAGATCAAGGTTGTTGGT	CAAAATTTGAGATCAAGGTTGTTGGC	GCAACCCTTAGTCACTTGATCTATCAATT
MU-Gm02-08	A	T	A	Gm02:44601276	CCACGAGTTGCCTAATGAAAAAAATTAC	ATTAACCACGAGTTGCCTAATGAAAAAAATTAT	CTCCTAACCCCCACGACATATAAATAATA
MU-Gm02-09	G	G	A	Gm02:44617603	TAATTATTCAATTAATGTATATAGAACATGCATG	ATTAATTATTCAATTAATGTATATAGAACATGCATA	CAGTTCAATTGTAAGGATAGTGATATACAT
MU-Gm02-10	G	K	T	Gm02:44671919	GCCAAACATTTTATTGTATATACATACACG	AAAGCCAAACATTTTATTGTATATACATACACT	GTGGAGCACCCTTTGCCCCAAT
MU-Gm02-11	T	T	C	Gm02:44671940	GCACCCTTTGCCCCAATACTCA	CACCCTTTGCCCCAATACTCG	GGCCACAATGCAAGAATCTATCAAGATAA
MU-Gm02-13	C	T	C	Gm02:44735733	TAATTAATTAAGATGAAGATGCTAAGACCGA	ATTAATTAAGATGAAGATGCTAAGACCGG	TTCATGCATCATAGGCCAATTAGGTTTGT
MU-Gm02-14	T	T	C	Gm02:44835549	TTAATACTACTTTTATGCTAGAGTAAGATAACA	ATACTACTTTTATGCTAGAGTAAGATAACG	ACAGCTTTAACCAGGTGAGAGTTAGAAA
MU-Gm02-15	C	C	T	Gm02:45035177	CTCAAGCTTAAGGATAGAGATTTTCCAAG	CTCAAGCTTAAGGATAGAGATTTTCCAAA	CCAAGTAGAAAGCCTCTCTGAGAGA
MU-Gm02-16	C	T	C	Gm02:45099312	TATTATAAAATTCAACAAACTTATCTGAATAAT	ATTATTATAAAATTCAACAAACTTATCTGAATAAC	GTTTGAAAAGAAATAGGTAATAGTTTGATA

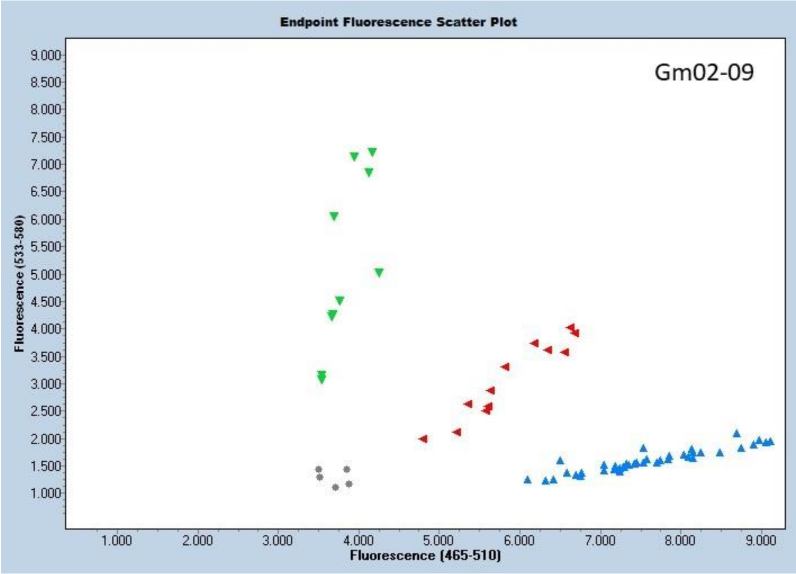

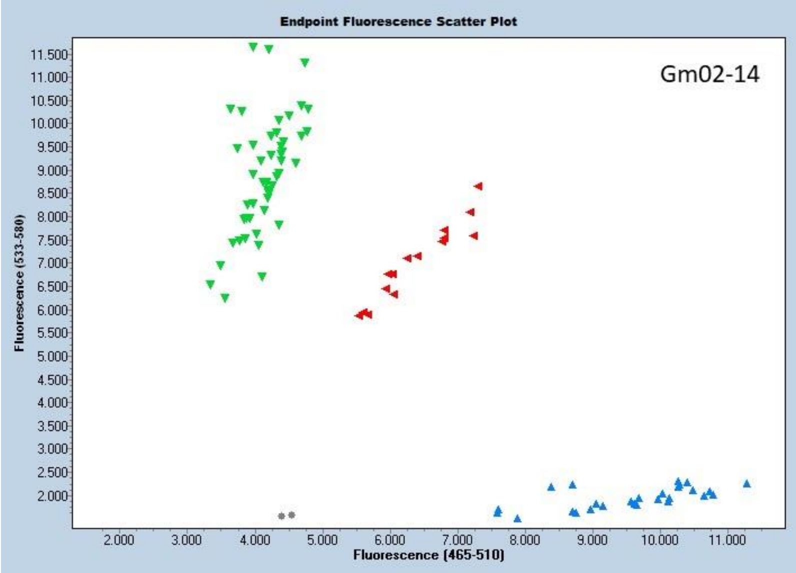

Supplementary Figure 8. (a) Fine-mapping of QTL02 in $F_{4:5}$ lines derived from heterozygous $F_{3:4}$ lines of PI 90763 x Peking. QTL02 has been fine-mapped to the region between markers Gm02-09 and Gm02-14. The 218 kb region (Gm02:44617603–44835549; Wm82.a2) contains 34 genes including *GmSNAP02* gene (*Glyma.02g260400*). All lines carry homozygous *rhg1-a*, *rhg2*, and *Rhg4*, but not QTL12. Black vertical lines signify recombination spots. The score between resistance (R) and moderate resistance (MR) was estimated based on standardized female index classification system (Schmitt and Shannon 1992) and confirmed with Tukey HSD test.

	Gm02_42012522_T_C	Gm02-09 (Gm02_44617603)	Gm02-14 (Gm02_44835549)	Gm02-15 (Gm02_45035177)	Gm02-16 (Gm02:45099312)	Gm02_46462690_T_C	NO. LINES	FI MIN	FI MAX	FI AV	SCORE
PI 90763	TT	AA	CC	TT	CC	TT	2	0	0.2	0.1	R
Peking	CC	GG	TT	CC	TT	CC	2	16	24	20	MR
19AS-84-5-74	CC	GG	TT	CC	TT	CC	4	14	15	14.4	MR
	CC	GG	TT	CC	TT	TT	2	15	16	15.5	MR
19AS-84-3-26	TT	AA	CC	TT	CC	CC	3	0	0.1	0	R
	TT	AA	CC	TT	CC	TT	4	0	0.1	0	R
19AS-84-2-95	TT	AA	CC	TT	CC	CC	5	0	0.6	0.2	R
	TT	AA	CC	TT	CC	TT	1	0	0	0	R
19AS-84-5-81	CC	GG	TT	CC	TT	CC	5	13	18	15	MR
	TT	AA	CC	TT	CC	CC	6	0	0.1	0	R
	CT	GA	TC	CT	TC	CC	3	1	6	3.2	R
19AS-84-2-97	CC	GG	TT	CC	TT	CC	6	15	22	17.9	MR
	TT	AA	CC	TT	TT	CC	2	0	0.2	0.1	R
	CT	GA	TC	CT	TT	CC	3	5	12	8.3	R
19AS-84-3-32	TT	GG	TT	CC	TT	CC	4	15	17	15.9	MR
	TT	GG	TT	TT	CC	TT	6	11	21	14.9	MR
19AS-84-5-80	CC	GG	CC	TT	CC	TT	3	10	14	11.7	MR
	TT	AA	CC	TT	CC	TT	3	0	0	0	R
19AS-84-3-37	TT	GG	TT	CC	TT	CC	5	14	15	14	MR
	TT	AA	CC	TT	CC	TT	4	0	0.2	0.1	R
19AS-84-5-92	CC	GG	TT	CC	TT	CC	2	10	16	13	MR
	CC	AA	CC	TT	CC	TT	5	0	0.2	0	R
	CC	GA	TC	CT	TC	CT	2	5	6	5.5	R
19AS-84-1-6	CC	GG	TT	CC	TT	CC	5	19	31	22.6	MR
	TT	AA	CC	TT	CC	TT	7	0	0.2	0.2	R
	CT	GA	TC	CT	TC	CT	3	2	7	3.8	R
19AS-84-3-54	CC	GG	TT	CC	TT	TT	5	15	17	16.2	MR
19AS-84-2-122	CC	GG	TT	CC	TT	CT	6	11	24	18	MR
19AS-84-1-9	CC	GG	TT	CC	TT	CT	3	14	17	15.5	MR
19AS-84-5-79	CC	AA	CC	TT	CC	TT	6	0	0.1	0	R
19AS-84-5-90	CC	AA	CC	TT	CC	TT	6	0	0.1	0.1	R
19AS-84-3-49	TT	GG	TT	CC	TT	CC	6	17	26	21.9	MR
19AS-84-2-114	CC	GG	TT	CC	TT	CC	6	18	23	20	MR

Supplementary Figure 8. (b) Thirty-four candidate genes in the 218 Kbp fine-mapped region (Gm02:44617603–44835549; Wm82.a2). *The GmSNAP02* gene (*Glyma.02g260400*) marked in red. Eleven genes highlighted in yellow represent a duplicated segment of chromosome 18 that includes the *GmSNAP18* gene.

Gene Model	Position (Wm82.v2.a1)	Protein/Function
Glyma.02g259100	Gm02_44623147-44626024	Plant protein of unknown function
Glyma.02g259200	Gm02_44628805-44629878	N/A
Glyma.02g259300	Gm02_44632522-44634801	Peroxidase
Glyma.02g259400	Gm02_44639898-44642041	Oligopeptide transporter-related protein
Glyma.02g259500	Gm02_44651207-44654419	Peroxidase
Glyma.02g259600	Gm02_44654567-44657064	N/A
Glyma.02g259700	Gm02_44658495-44659805	Hypoxia-responsive family protein
Glyma.02g259800	Gm02_44662823-44664812	N/A
Glyma.02g259900	Gm02_44675719-44677278	N/A
Glyma.02g260000	Gm02_44682536-44683450	Protein of unknown function
Glyma.02g260100	Gm02_44684684-44688689	Transmembrane amino acid transporter protein
Glyma.02g260200	Gm02_44688786-44689712	Transmembrane amino acid transporter protein
Glyma.02g260300	Gm02_44689952-44693546	Transmembrane amino acid transporter protein
Glyma.02g260400	Gm02_44695637-44698195	Alpha-soluble NSF attachment protein
Glyma.02g260500	Gm02_44701769-44704798	Protein of unknown function
Glyma.02g260600	Gm02_44706034-44708734	Staphylococcal nuclease homologue
Glyma.02g260700	Gm02_44711909-44712658	Copper chaperone
Glyma.02g260800	Gm02_44714204-44715022	N/A
Glyma.02g260900	Gm02_44716758-44717484	Arogenate dehydrogenase
Glyma.02g261000	Gm02_44718291-44721287	N/A
Glyma.02g261100	Gm02_44726522-44728449	Prenylated rab acceptor 1
Glyma.02g261200	Gm02_44740164-44742226	Cell division cycle protein D123
Glyma.02g261300	Gm02_44740814-44741095	Cell division cycle protein D123
Glyma.02g261400	Gm02_44744143-44747306	Leucine-rich repeat receptor-like protein kinase
Glyma.02g261500	Gm02_44747676-44753250	Sorting nexin
Glyma.02g261600	Gm02_44758969-44763198	Multicopper oxidase
Glyma.02g261700	Gm02_44767828-44776703	AP2 DNA-binding domain
Glyma.02g261800	Gm02_44776099-44783352	DNA repair protein, helicase
Glyma.02g261900	Gm02_44792699-44793865	Polyketide cyclase / dehydrase and lipid transport
Glyma.02g262000	Gm02_44798967-44799846	N/A
Glyma.02g262100	Gm02_44802750-44805813	GDSL/SGNH-like Acyl-Esterase family
Glyma.02g262200	Gm02_44812653-44814835	Glycosyl hydrolase family 85
Glyma.02g262300	Gm02_44820777-44831595	Glycosyl hydrolase family 85
Glyma.02g262400	Gm02_44834256-44835310	Protein of unknown function

WHOLE GENOME SEQUENCING READS SUPPLEMENTARY DATA

Supplementary Figure 9. Soybean Allele Catalog (https://www.soykb.org) displaying 11 variant alleles of the *GmSNAP02* gene. PI 90763 and Peking (PI 548402) displays the same allele as Williams 82 reference genome (Wm82.a2). Red font in PI 90763 indicates imputed variants.

Soja	Landrace	Elite	Total	Cultivar	44695733	44695753	44695865	44695950	44695972	44696001	44696179	44696184	44697215	44697321	44697698	44697700
34	221	238	548	87	AATATGGGCG|Ref	GAGGGCCGAGGAT TTTGAGAACA|Alt	A|Ref	AG|Ref	TAC|Ref	GGTAAC|Ref	A|Ref	T|Ref	C|Ref	T|Ref	CT|Ref	G|Ref
72	231	141	497	51	AATATGGGCG|Ref	CAGGGCCGAGGAT TTTGAGAACA|Ref	A|Ref	AG|Ref	TAC|Ref	GGTAAC|Ref	A|Ref	T|Ref	C|Ref	T|Ref	CT|Ref	G|Ref
0	5	2	7	2	AATATGGGCG|Ref	C|frameshift_variant|A8fs	A|Ref	AG|Ref	TAC|Ref	GGTAAC|Ref	A|Ref	T|Ref	C|Ref	T|Ref	CT|Ref	G|Ref
0	4	0	4	0	AATATGGGCG|Ref	CAGGGCCGAGGAT TTTGAGAACA|Ref	A|Ref	AG|Ref	T|frameshift_variant|I57fs	GGTAAC|Ref	A|Ref	T|Ref	C|Ref	T|Ref	CT|Ref	G|Ref
2	0	0	2	0	AATATGGGCG|Ref	CAGGGCCGAGGAT TTTGAGAACA|Ref	A|Ref	A|splice_acceptor_variant&intron_variant	TAC|Ref	GGTAAC|Ref	A|Ref	T|Ref	C|Ref	T|Ref	CT|Ref	G|Ref
2	0	0	2	0	AATATGGGCG|Ref	CAGGGCCGAGGAT TTTGAGAACA|Ref	A|Ref	AG|Ref	TAC|Ref	GGTAAC|Ref	AAT|frameshift_variant&splice_region_variant|E90fs	T|Ref	C|Ref	T|Ref	CT|Ref	G|Ref
1	0	0	1	0	AATATGGGCG|Ref	CAGGGCCGAGGAT TTTGAGAACA|Ref	A|Ref	AG|Ref	TAC|Ref	GGTAAC|Ref	A|Ref	A|splice_donor_variant&intron_variant	C|Ref	T|Ref	CT|Ref	G|Ref
0	1	0	1	0	AATATGGGCG|Ref	CAGGGCCGAGGAT TTTGAGAACA|Ref	T|K44*	AG|Ref	TAC|Ref	GGTAAC|Ref	A|Ref	T|Ref	C|Ref	T|Ref	CT|Ref	G|Ref
0	1	0	1	0	AATATGGGCG|Ref	CAGGGCCGAGGAT TTTGAGAACA|Ref	A|Ref	AG|Ref	TAC|Ref	G|splice_donor_variant&splice_region_variant&intron_variant	A|Ref	T|Ref	C|Ref	T|Ref	CT|Ref	G|Ref
0	1	0	1	0	AATATGGGCG|Ref	CAGGGCCGAGGAT TTTGAGAACA|Ref	A|Ref	AG|Ref	TAC|Ref	GGTAAC|Ref	A|Ref	T|Ref	C|Ref	T|Ref	C|frameshift_variant|E252fs	A|E252K
0	1	0	1	0	AATATGGGCG|Ref	CAGGGCCGAGGAT TTTGAGAACA|Ref	A|Ref	AG|Ref	TAC|Ref	GGTAAC|Ref	A|Ref	T|Ref	C|Ref	C|L204P	CT|Ref	G|Ref
0	1	0	1	0	AATATGGGCG|Ref	CAGGGCCGAGGAT TTTGAGAACA|Ref	A|Ref	AG|Ref	TAC|Ref	GGTAAC|Ref	A|Ref	T|Ref	T|Q169*	T|Ref	CT|Ref	G|Ref

	44695733	44695753	44695865	44695950	44695972	44696001	44696179	44696184	44697215	44697321	44697698	44697700	Imputation
PI 90763 (USB-054_PI090763)	AATATGGGCG|Ref	CAGGGCCGAGGAT TTTGAGAACA|Ref	A|Ref	AG|Ref	TAC|Ref	GGTAAC|Ref	A|Ref	T|Ref	C|Ref	T|Ref	CT|Ref|+	G|Ref|+	+
Peking (USB-027_PI548402)	AATATGGGCG|Ref	CAGGGCCGAGGAT TTTGAGAACA|Ref	A|Ref	AG|Ref	TAC|Ref	GGTAAC|Ref	A|Ref	T|Ref	C|Ref	T|Ref	CT|Ref	G|Ref	-

Supplementary Figure 10. JBrowse output of copy number variation (CNV) of *GmSNAP02* gene (*Glyma.02G260400*): (a) CNV was unlikely at *GmSNAP02* in PI 90763; (b) Internal validation of CNV at *Rhg1* confirmed different copy numbers at *GmSNAP18* between PI 88788 and PI 90763.

a

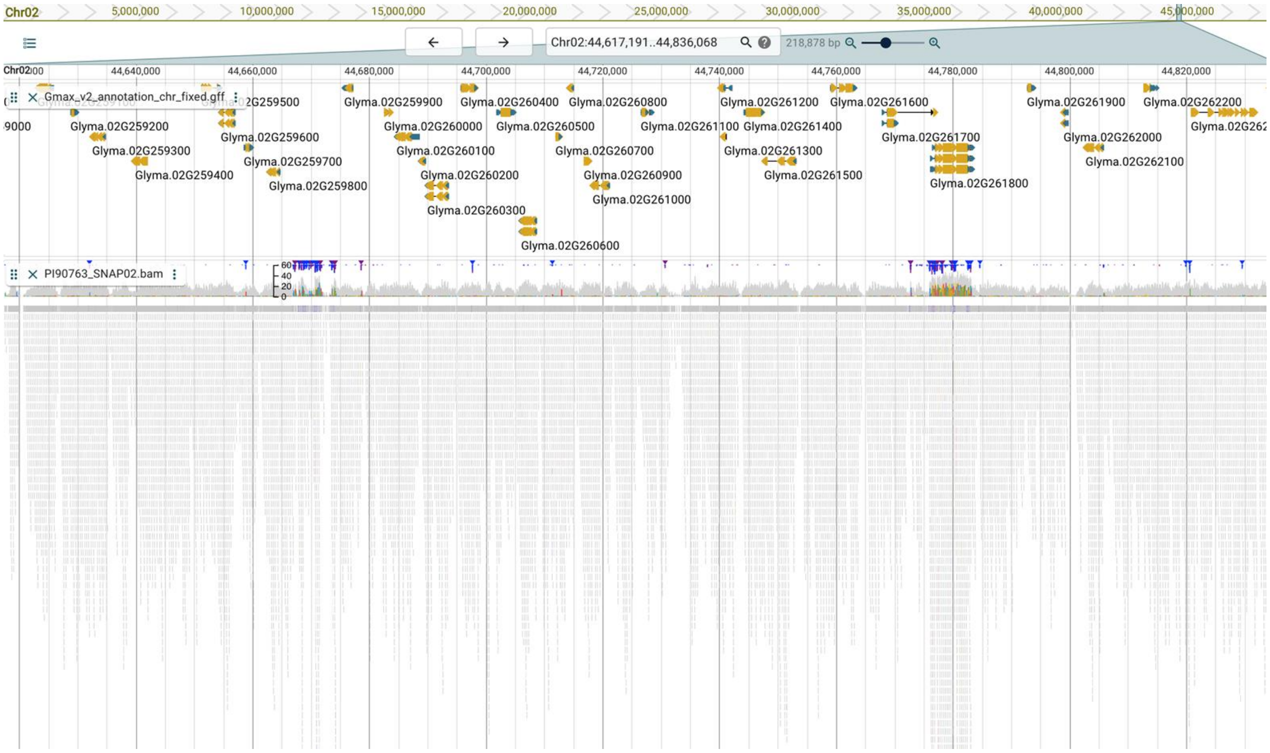

b

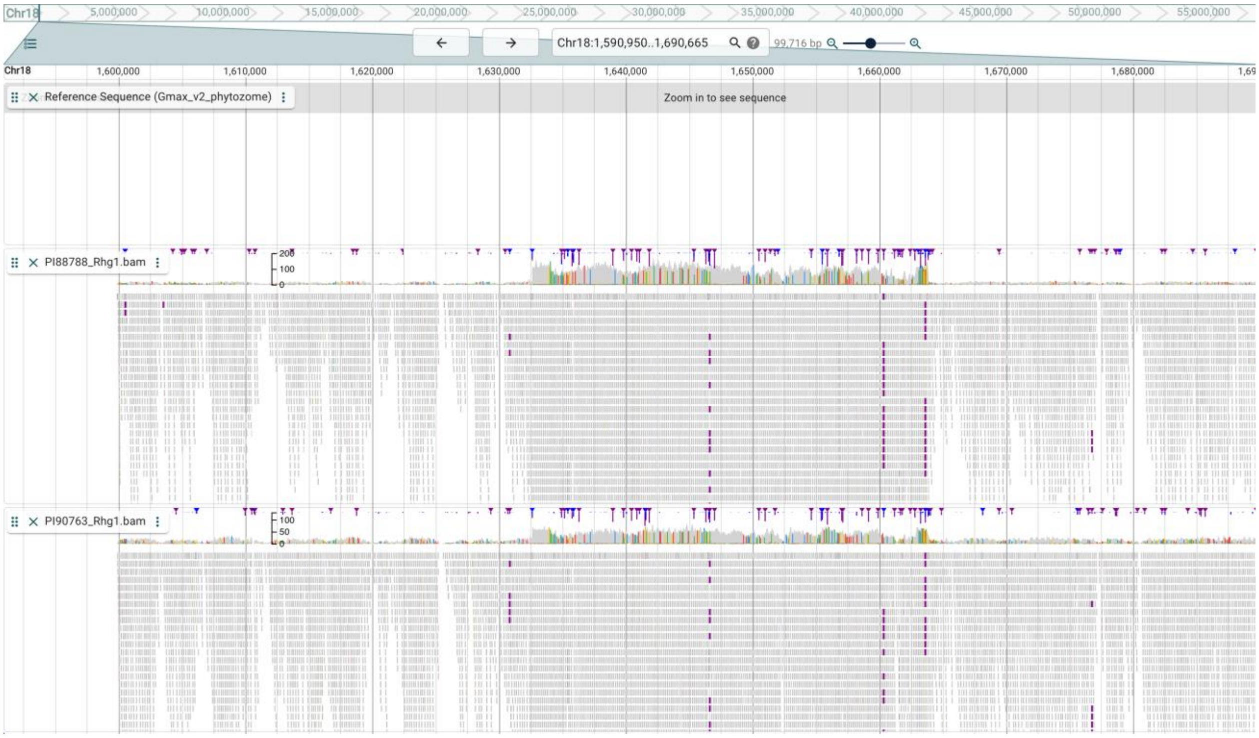

Supplementary Figure 11. Whole genome resequencing-derived variant calls in PI 90763, Peking, PI 437654, and Williams 82 across the *GmSNAP02* gene: (a) A pattern of reads with poor alignment (multicolored) in the eighth exon of the gene in PI 90763, and 22 nt deletion in exon 1 in PI 437654. The dashed box signifies the place of the insertion in PI 90763 and deletion in PI 437654. The boxes on the left show enlarged view. (b) hypothesis of how a putative insertion in exon 8 of PI 90763 could be excluded from the alignment data.

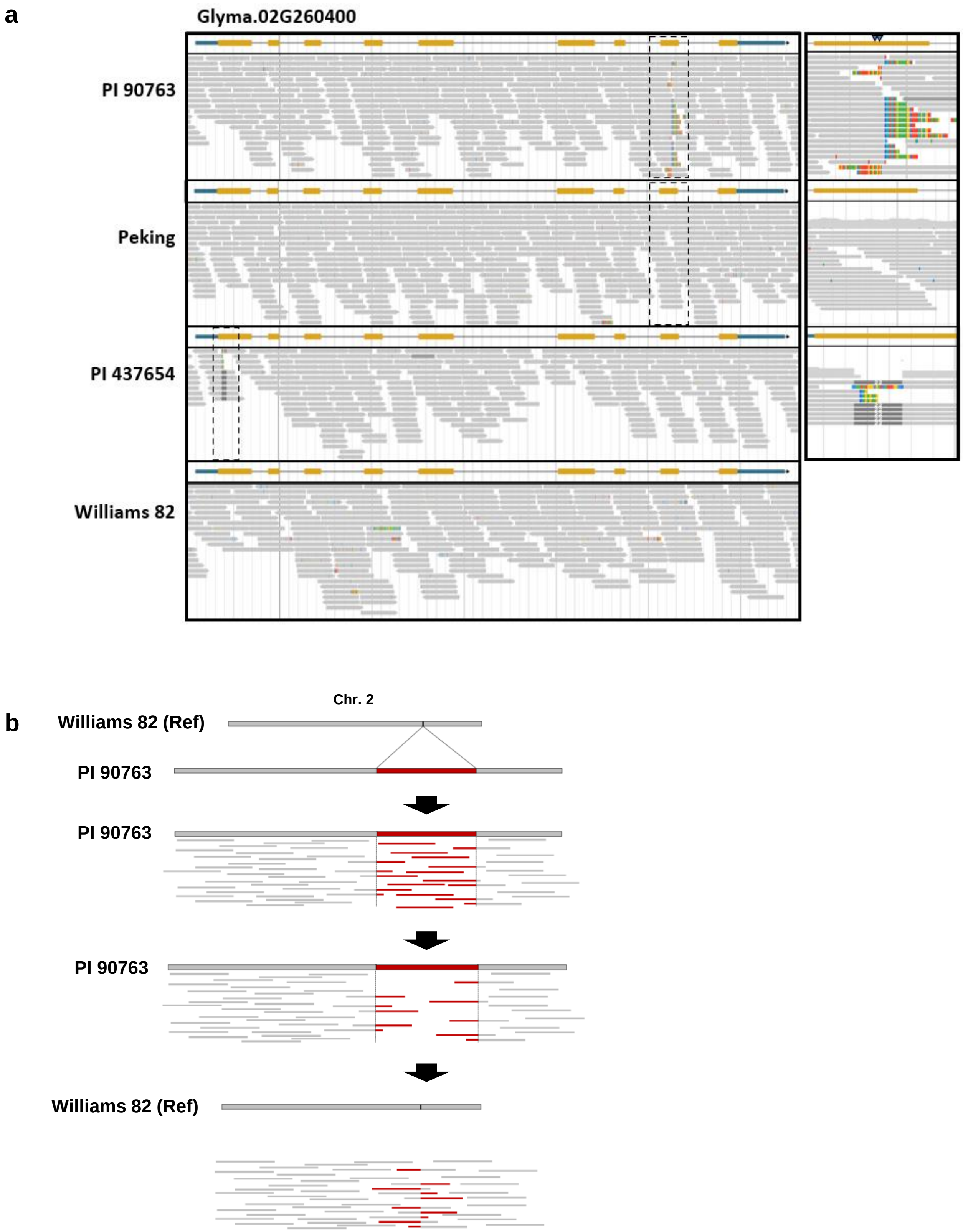

Supplementary Figure 12. Summary of primers used in this research.

Purpose	Primer name	Sequence 5'-3'
Amplify the fragment flanking the insertion of *GmSNAP02* from gDNA	F1	CTGACCTTCCTGTGTCAC
	R1	ACCGAAAGAAGACCATGG
To amplify full length *GmSNAP02* transcript from cDNA	F2	ATGAAAGAGAGAGCATTAAATTGAATTG
	R2	CCAACCGAAAGAAGACCATGG
GmSNAP02 qRT-PCR primers	qRT-PCR - F	TTGTACGAGTCTGAACAGAATATCTC
	qRT-PCR - R	CTCAATTGATCTCTGATATTGTTCA
Genotyping *GmSNAP02* T4+T3 edited roots	F3	ATGAAAGAGAGAGCATTAAATTGAATTG
	R3	GCAGATGTTCATGCATTATTATC
Genotyping *GmSNAP02* T5+T7 edited roots	F4	GGTCGCGGTAATTAATCAACAT
	R4	TCCAACAGCTATTTCACTTGTCA
To check off-targets in *GmSNAP02*-T4+T3 transformed roots	F5	CATAGATTGCATGAAAGAGAGGG
	R5	CCAACAAACATAGAACAATAGAAG
To check off-targets in *GmSNAP02*-T5+T7 transformed roots	F6	GCATGTTTGCGTGAATGTTG
	R6	CTCACTCGTCTACTTAAGCAAGC
Primers to generate gRNA fragments	T3 - F	GATTGTCTGAACAGAATATCTCGC
	T3 - R	AAACGCGAGATATTCTGTTCAGAC
	T4 - F	GATTGTATGGGCGATCATTTGGCCA
	T4 - R	AAACTGGCCAAATGATCGCCCATAC
	T5 - F	GATTGACTCAAAGCCAAAGAAATCG
	T5 - R	AAACCGATTTCTTTGGCTTTGAGTC
	T7 - F	GATTGATCCAACCGAAAGAAGACCA
	T7 - R	AAACTGGTCTTCTTTCGGTTGGATC
Primers for sequencing the Atu6-gDNA constructs in AtU6-26SK	M13R	CACAGGAAACAGCTATGAC
	SS42 (Sequencing)	TCCCAGGATTAGAATGATTAGG
Primers to check final vector construct by PCR	SS42	TCCCAGGATTAGAATGATTAGG
	Cas9 - F	GCCCAAGAGGAACAGAGTAAGC
	Cas9 - R	CAGTTCGCCGGCAGAGGCCAGC

Supplementary Figure 13. (a) Alignment of full-length cDNA sequences of *GmSNAP02* from Williams 82, Peking, and PI 437654. The cDNA sequences of *GmSNAP02* from Williams 82 and Peking were identical, while PI 437654 had a 22 nt deletion at the 5' end.

```
Wm82          ATGGGCGATCATTTGGCCAGGGCCGAGGATTTTGAGAACAAGGCAGAGAAGAAACTCAGC   60
Peking        ATGGGCGATCATTTGGCCAGGGCCGAGGATTTTGAGAACAAGGCAGAGAAGAAACTCAGC   60
PI437654      ATGGGCGATCATTTGGCC----------------------AGGCAGAGAAGAAACTCAGC   38
              ******************                      ********************

Wm82          AGTTGGGGCTTGTTTGGCTCCAAATTCGAGGACGCTGCTGATCTCTTCGACAAATCCGCC   120
Peking        AGTTGGGGCTTGTTTGGCTCCAAATTCGAGGACGCTGCTGATCTCTTCGACAAATCCGCC   120
PI437654      AGTTGGGGCTTGTTTGGCTCCAAATTCGAGGACGCTGCTGATCTCTTCGACAAATCCGCC   98
              ************************************************************

Wm82          AATTCCTATAAGCTCGCTAAATCATGGGACAAAGCAGGATCCACCTACATCAAATTAGCG   180
Peking        AATTCCTATAAGCTCGCTAAATCATGGGACAAAGCAGGATCCACCTACATCAAATTAGCG   180
PI437654      AATTCCTATAAGCTCGCTAAATCATGGGACAAAGCAGGATCCACCTACATCAAATTAGCG   158
              ************************************************************

Wm82          AGTTGTCATTTGAAGTTGGAAAGCAAGCATGAAGCTGCACAAGCTTATGTTGACGCTGCG   240
Peking        AGTTGTCATTTGAAGTTGGAAAGCAAGCATGAAGCTGCACAAGCTTATGTTGACGCTGCG   240
PI437654      AGTTGTCATTTGAAGTTGGAAAGCAAGCATGAAGCTGCACAAGCTTATGTTGACGCTGCG   218
              ************************************************************

Wm82          CGTTGCTATAAAAAAACTAATATAAATGAGTCTGTATCTTGCTTAGACAATGCTGTAAAT   300
Peking        CGTTGCTATAAAAAAACTAATATAAATGAGTCTGTATCTTGCTTAGACAATGCTGTAAAT   300
PI437654      CGTTGCTATAAAAAAACTAATATAAATGAGTCTGTATCTTGCTTAGACAATGCTGTAAAT   278
              ************************************************************

Wm82          ATTTTCTGTGAGATTGGAAGACTCTCTATGGCTGCTAGATATTTGAAGGAAATTGCTGAG   360
Peking        ATTTTCTGTGAGATTGGAAGACTCTCTATGGCTGCTAGATATTTGAAGGAAATTGCTGAG   360
PI437654      ATTTTCTGTGAGATTGGAAGACTCTCTATGGCTGCTAGATATTTGAAGGAAATTGCTGAG   338
              ************************************************************

Wm82          TTGTACGAGTCTGAACAGAATATCTCGCAGGCCGTTGCTTACTATGAAAAATCAGCGGAT   420
Peking        TTGTACGAGTCTGAACAGAATATCTCGCAGGCCGTTGCTTACTATGAAAAATCAGCGGAT   420
PI437654      TTGTACGAGTCTGAACAGAATATCTCGCAGGCCGTTGCTTACTATGAAAAATCAGCGGAT   398
              ************************************************************

Wm82          TTTTTTGAAAATGAAGAAGTGAACACTTCAGCAAACCAGTGCAAGCAAAAAGTTGCTCAA   480
Peking        TTTTTTGAAAATGAAGAAGTGAACACTTCAGCAAACCAGTGCAAGCAAAAAGTTGCTCAA   480
PI437654      TTTTTTGAAAATGAAGAAGTGAACACTTCAGCAAACCAGTGCAAGCAAAAAGTTGCTCAA   458
              ************************************************************

Wm82          TTCTCTGCCCAGCTTGAACAATATCAGAGATCAATTGAGATTTATGAAGATATTGCTCGC   540
Peking        TTCTCTGCCCAGCTTGAACAATATCAGAGATCAATTGAGATTTATGAAGATATTGCTCGC   540
PI437654      TTCTCTGCCCAGCTTGAACAATATCAGAGATCAATTGAGATTTATGAAGATATTGCTCGC   518
              ************************************************************

Wm82          CAGTCTCTCAGCAATACTTTGCTGAAGTATGGAGTTAAAGGGCATCTTCTTAATGCTGGC   600
Peking        CAGTCTCTCAGCAATACTTTGCTGAAGTATGGAGTTAAAGGGCATCTTCTTAATGCTGGC   600
PI437654      CAGTCTCTCAGCAATACTTTGCTGAAGTATGGAGTTAAAGGGCATCTTCTTAATGCTGGC   578
              ************************************************************

Wm82          ATTTGCGAACTTTGTAAAGGGGATGTTATTGCTATTACCAATGCATTGGAGCGATATCAG   660
Peking        ATTTGCGAACTTTGTAAAGGGGATGTTATTGCTATTACCAATGCATTGGAGCGATATCAG   660
PI437654      ATTTGCGAACTTTGTAAAGGGGATGTTATTGCTATTACCAATGCATTGGAGCGATATCAG   638
              ************************************************************

Wm82          GACTTGGATCCAACATTTTCTGGAACACGTGAATATAGACTTCTGGCAGATATTGCTGCT   720
Peking        GACTTGGATCCAACATTTTCTGGAACACGTGAATATAGACTTCTGGCAGATATTGCTGCT   720
PI437654      GACTTGGATCCAACATTTTCTGGAACACGTGAATATAGACTTCTGGCAGATATTGCTGCT   698
              ************************************************************

Wm82          GCAATTGATGAGGAAGATGTTGGAAAGTTTACTGAAGTTATCAAGGAATTTGATAGTTTG   780
Peking        GCAATTGATGAGGAAGATGTTGGAAAGTTTACTGAAGTTATCAAGGAATTTGATAGTTTG   780
PI437654      GCAATTGATGAGGAAGATGTTGGAAAGTTTACTGAAGTTATCAAGGAATTTGATAGTTTG   758
              ************************************************************

Wm82          ACTCCTTTGGATTCTTGGAAGACAACACTTCTTTTGAGGGTGAAAGATAAACTCAAAGCC   840
Peking        ACTCCTTTGGATTCTTGGAAGACAACACTTCTTTTGAGGGTGAAAGATAAACTCAAAGCC   840
PI437654      ACTCCTTTGGATTCTTGGAAGACAACACTTCTTTTGAGGGTGAAAGATAAACTCAAAGCC   818
              ************************************************************

Wm82          AAAGAAATCGAGGAGGATGATCTTACTTGA                                 870
Peking        AAAGAAATCGAGGAGGATGATCTTACTTGA                                 870
PI437654      AAAGAAATCGAGGAGGATGATCTTACTTGA                                 848
              ******************************
```

Supplementary Figure 13. (b) Alignment of amino acid sequences of GmSNAP02 from Williams 82, Peking, and PI 437654. The 22 nt deletion at the 5' end of *GmSNAP02* in PI 437654 causes a frameshift mutation resulting in a premature stop codon in the amino acid sequence.

```
Wm82            MGDHLARAEDFENKAEKKLSSWGLFGSKFEDAADLFDKSANSYKLAKSWDKAGSTYIKLA    60
Peking          MGDHLARAEDFENKAEKKLSSWGLFGSKFEDAADLFDKSANSYKLAKSWDKAGSTYIKLA    60
PI 437654       MGDHLARQRRNSAV----------------------------------------------    14
                ******* .  .

Wm82            SCHLKLESKHEAAQAYVDAARCYKKTNINESVSCLDNAVNIFCEIGRLSMAARYLKEIAE    120
Peking          SCHLKLESKHEAAQAYVDAARCYKKTNINESVSCLDNAVNIFCEIGRLSMAARYLKEIAE    120
PI 437654       ------------------------------------------------------------    14

Wm82            LYESEQNISQAVAYYEKSADFFENEEVNTSANQCKQKVAQFSAQLEQYQRSIEIYEDIAR    180
Peking          LYESEQNISQAVAYYEKSADFFENEEVNTSANQCKQKVAQFSAQLEQYQRSIEIYEDIAR    180
PI 437654       ------------------------------------------------------------    14

Wm82            QSLSNTLLKYGVKGHLLNAGICELCKGDVIAIT--N------ALERYQDLDPTFSGTREY    232
Peking          QSLSNTLLKYGVKGHLLNAGICELCKGDVIAIT--N------ALERYQDLDPTFSGTREY    232
PI 437654       -------------------GACLAPNSRTLLISSTNPPIPISSLNHGTKQDPPTSN----    51
                                   * *   :. .: *:  *      :*::  . **  *.

Wm82            RLLADIAAAIDEEDVGKFTEVIKEFDSLTPLDSWKTTLLLRVKDKLKAKEIEEDDLT  289
Peking          RLLADIAAAIDEEDVGKFTEVIKEFDSLTPLDSWKTTLLLRVKDKLKAKEIEEDDLT  289
PI 437654       ---------------------------------------------------------  51
```

EXPRESSION & CRISPR-CAS9 OF *GmSNAP02* SUPPLEMENTARY DATA

Supplementary Figure 14. Characterization of $F_{4:5}$ recombinant sister lines 19AS-84-5-81-8 (81-8) and 19AS-84-5-81-4 (81-4) used for expression studies.

Line	Pedigree	*GmSNAP18-a*	*GmSNAP11*	*GmSHMT08*	*GmSNAP02-ins*	QTL border (Gm02:42012522)	Gm02-09 (Gm02:44617603)	Gm02-14 (Gm02:44835549)	Gm02-15 (Gm02:45035177)	Gm02-16 (Gm02:45099312)	QTL border (Gm02:46462690)
19AS-84-5-81-8	PI 90763 × Peking	✓	✓	✓	✓	T	Allele Y	Allele Y	Allele Y	Allele Y	C
19AS-84-5-81-4	PI 90763 × Peking	✓	✓	✓	×	C	Allele X	Allele X	Allele X	Allele X	C
PI 90763	N/A	✓	✓	✓	✓	T	Allele Y	Allele Y	Allele Y	Allele Y	T
Peking	N/A	✓	✓	✓	×	C	Allele X	Allele X	Allele X	Allele X	C

Line	Pedigree	TN22 (HG type 1.2.5.7; Race 2)						
		Rep 1	Rep 2	Rep 3	Rep 4	Rep 5	MEAN	FI
19AS-84-5-81-8	PI 90763 X Peking	0	0	0	1	0	0.2	0
19AS-84-5-81-4	PI 90763 X Peking	40	37	57	34	38	41.2	16
PI 90763	N/A	0	1	1	0	0	0.4	0
Peking	N/A	26	35	45	59	38	40.6	16

Line	Pedigree	MM26 (HG type 1.2.5.7; Race 2)											
		Rep 1	Rep 2	Rep 3	Rep 4	Rep 5	Rep 6	Rep 7	Rep 8	Rep 9	Rep 10	MEAN	FI
19AS-84-5-81-8	PI 90763 X Peking	2	5	5	2	3	2	0	0	1	1	2.1	1
19AS-84-5-81-4	PI 90763 X Peking	17	16	25	15	12	12	14	15	10	17	15.3	9
PI 90763	N/A	0	0	0	0	0	1	0	0	0	1	0.2	0
Peking	N/A	30	21	17	14	24	26	28	21	34	16	23.1	14

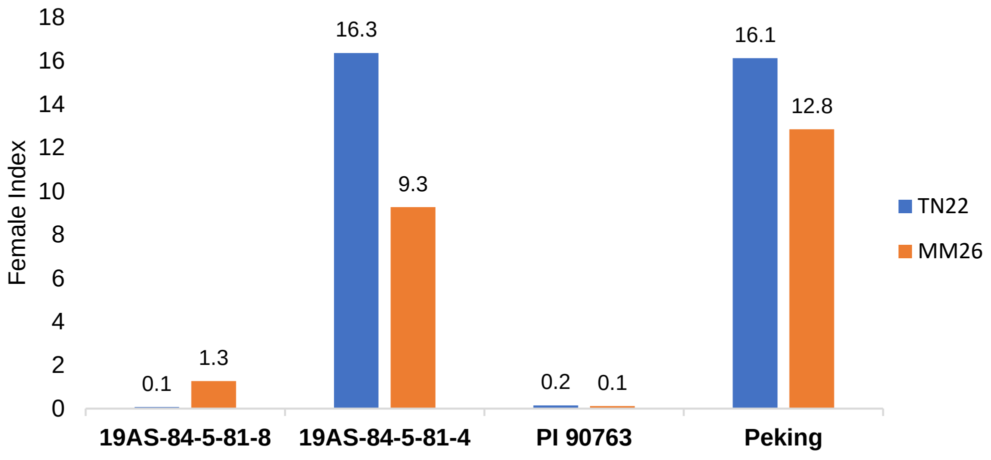

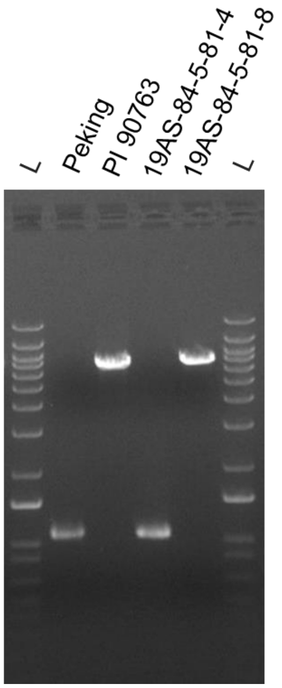

Supplementary Figure 15. Biological replicate 1. Peking roots transformed with CRISPR/Cas9-EV, CRISPR/Cas9-*GmSNAP02*-T4+T3, and CRISPR/Cas9-*GmSNAP02*-T5+T7 under fluorescent light. Fourteen plants with GFP-positive roots for each construct were selected for phenotyping assays. Cyst counts for each plant are indicated below the corresponding plant. Pictures taken just before transplanting and nematode inoculation.

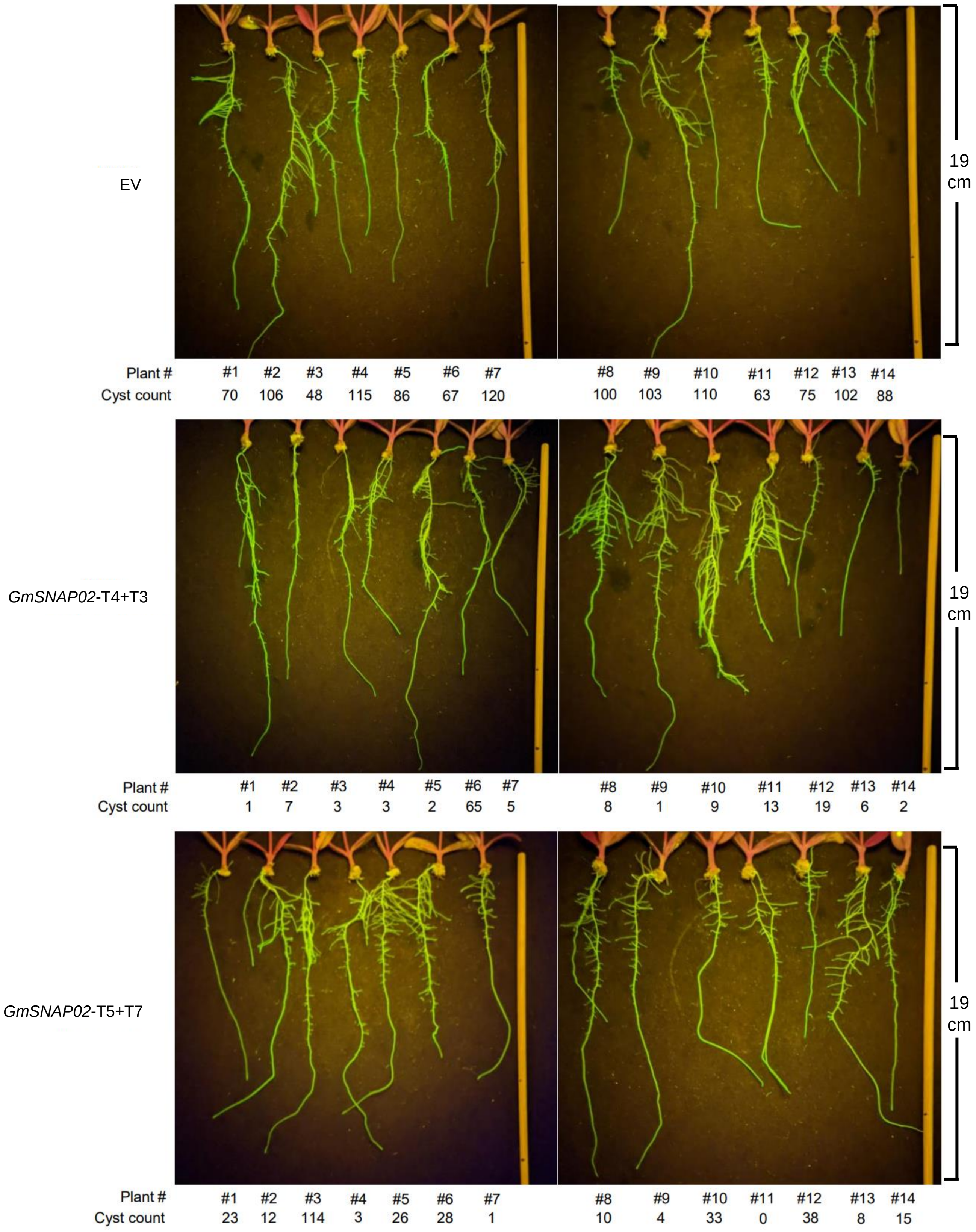

Supplementary Figure 16. Biological replicate 1. PI 90763 roots transformed with CRISPR/Cas9-EV, CRISPR/Cas9-*GmSNAP02*-T4+T3, and CRISPR/Cas9-*GmSNAP02*-T5+T7 under fluorescent light. Fourteen plants with GFP-positive roots for each construct were selected for phenotyping assays. Cyst counts for each plant are indicated below the corresponding plant. Pictures taken just before transplanting and nematode inoculation.

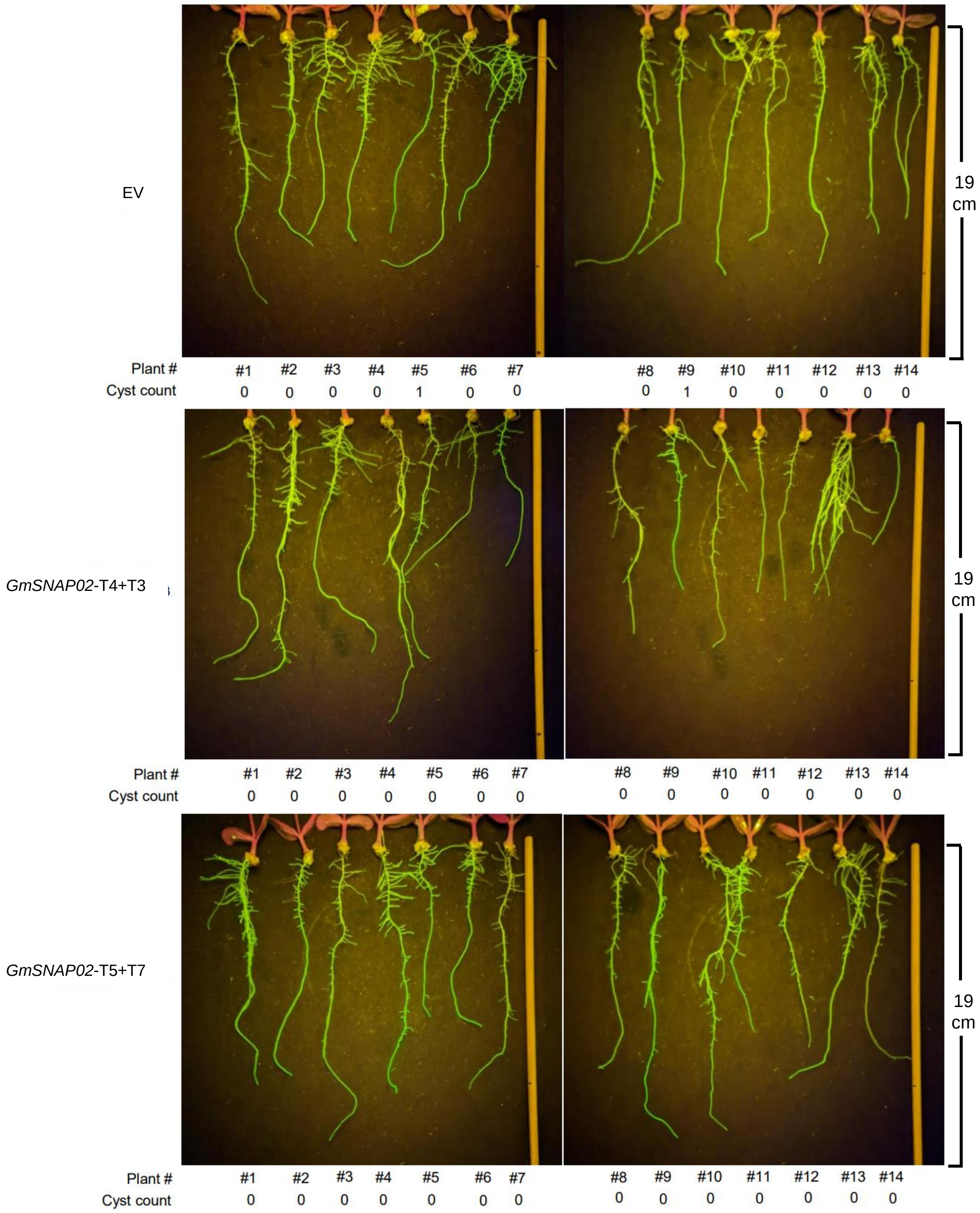

Supplementary Figure 17. Biological replicate 2. Peking roots transformed with CRISPR/Cas9-EV, CRISPR/Cas9-*GmSNAP02*-T4+T3, and CRISPR/Cas9-*GmSNAP02*-T5+T7 under fluorescent light. Fourteen plants with GFP-positive roots for each construct were selected for phenotyping assays. Cyst counts for each plant are indicated below the corresponding plant. Pictures taken just before transplanting and nematode inoculation.

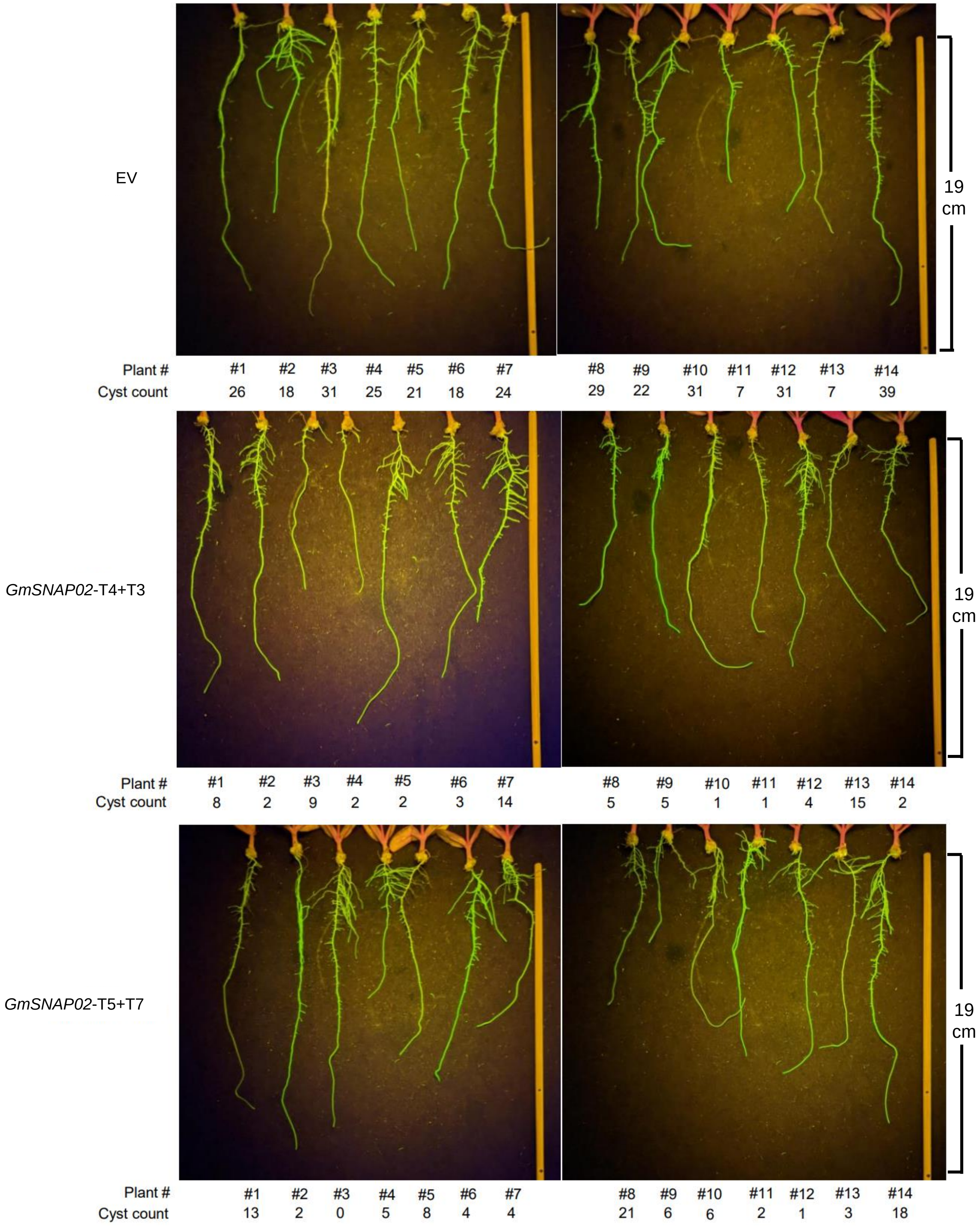

Supplementary Figure 18. Biological replicate 2. PI 90763 roots transformed with CRISPR/Cas9-EV, CRISPR/Cas9-*GmSNAP02*-T4+T3, and CRISPR/Cas9-*GmSNAP02*-T5+T7 under fluorescent light. Fourteen plants with GFP-positive roots for each construct were selected for phenotyping assays. Cyst counts for each plant are indicated below the corresponding plant. Pictures taken just before transplanting and nematode inoculation.

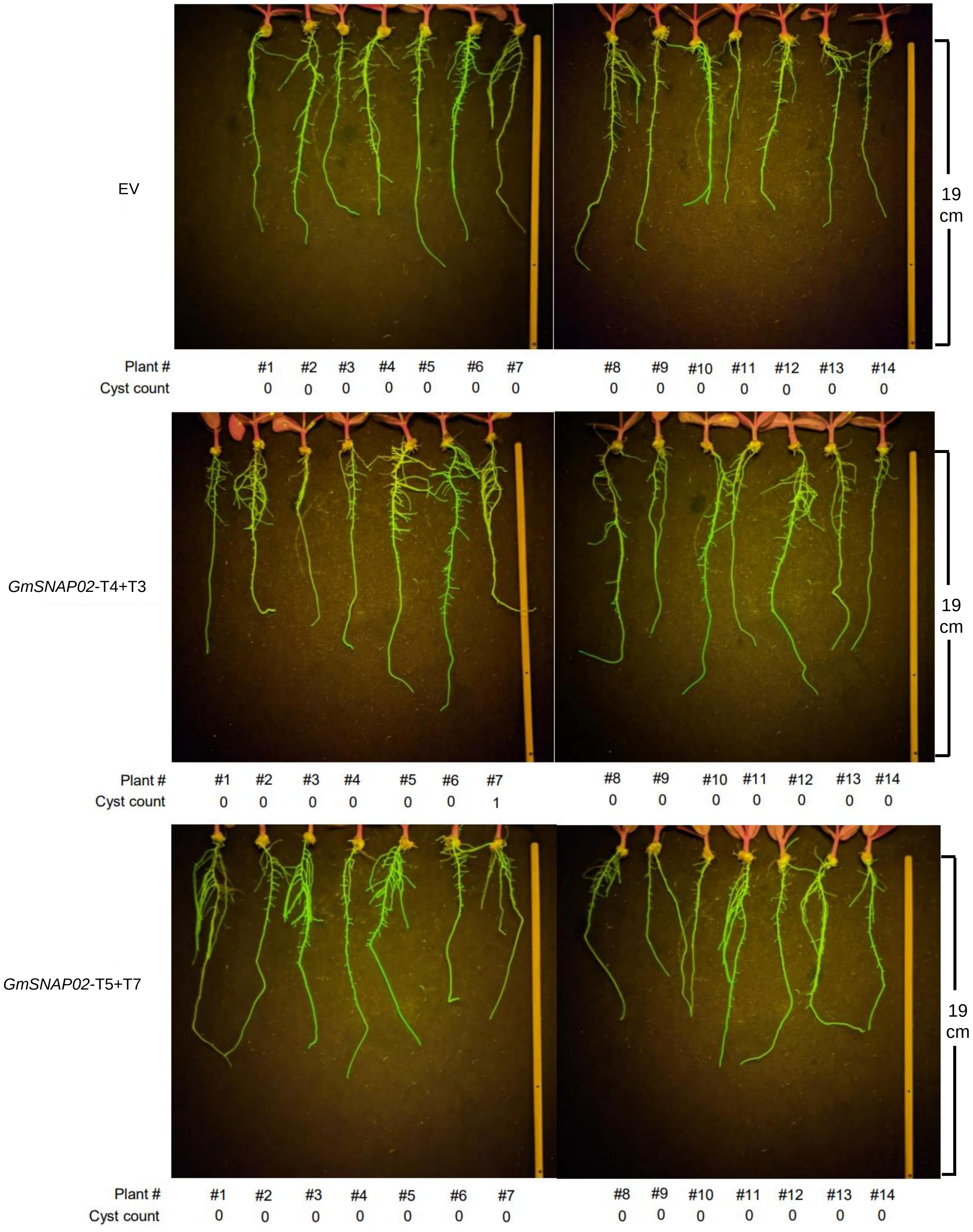

Supplementary Figure 19. Targeted genome editing of *GmSNAP02* in Peking and PI 90763 by CRISPR/Cas9-*GmSNAP02*-T4+T3 dual gRNA system. Genomic DNA was extracted from transgenic hairy roots and the targeted fragments were amplified by PCR. Direct sequencing of PCR products confirmed successful gene edits, as indicated by the presence of deletions/insertions and re-annealing at specific positions (marked by red triangles on the chromatograms).

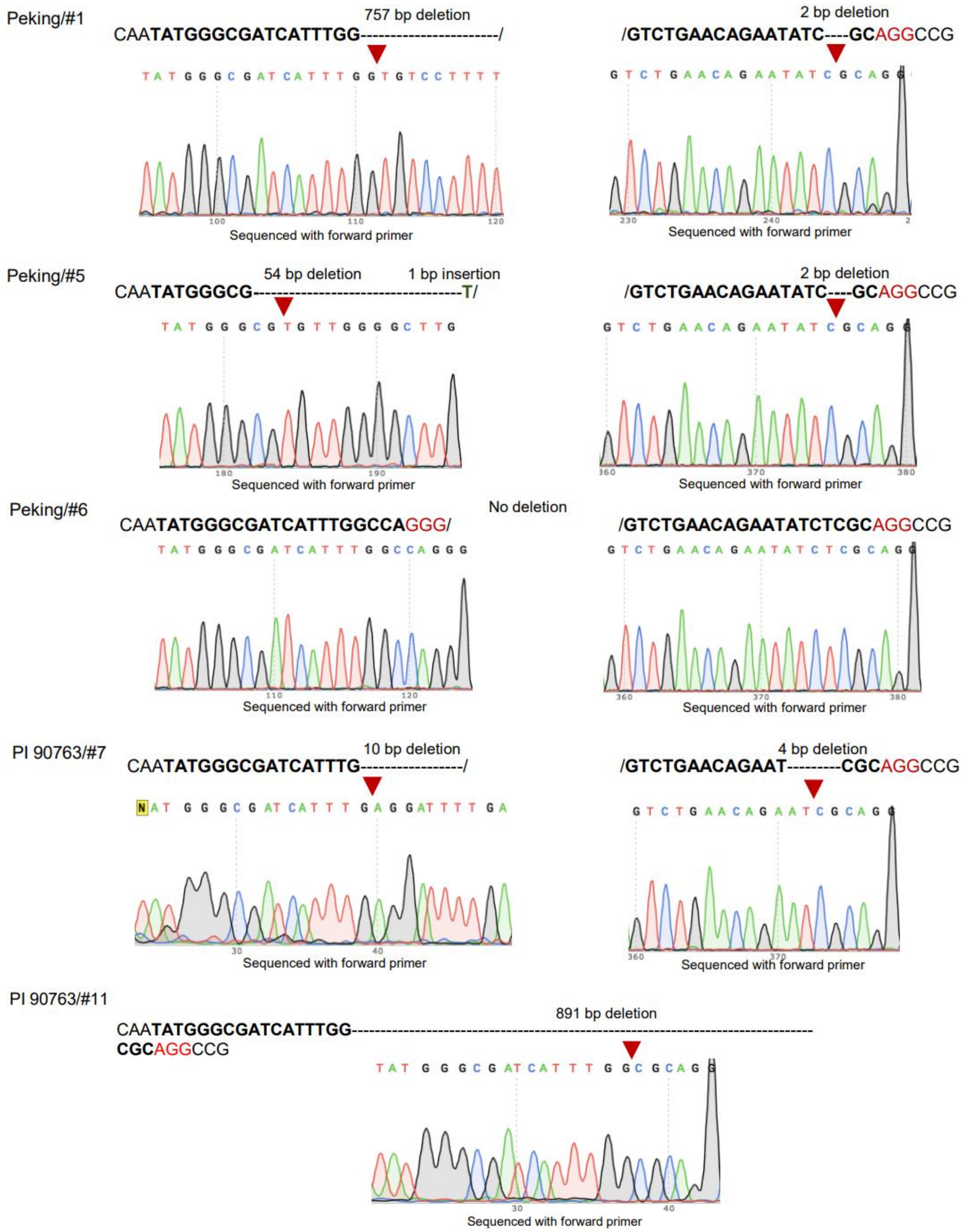

Supplementary Figure 20. Targeted genome editing of *GmSNAP02* in Peking and PI 90763 by CRISPR/Cas9-*GmSNAP02*-T5+T7 dual gRNA system. Genomic DNA was extracted from transgenic hairy roots, and the targeted fragments were amplified by PCR. Direct sequencing of PCR products confirmed successful gene edits, as indicated by the presence of deletions/insertions or chimeric sequences at specific positions (marked by red triangles on the chromatograms). There was no deletion in *GmSNAP02* in Peking/#3. Peking/#4 and Peking/#10 displayed complete deletion and insertions between the target sites. Similarly, the chromatogram of PI 90763 #7 and #9 showed overlapping traces, confirming successful gene editing in the targeted region.

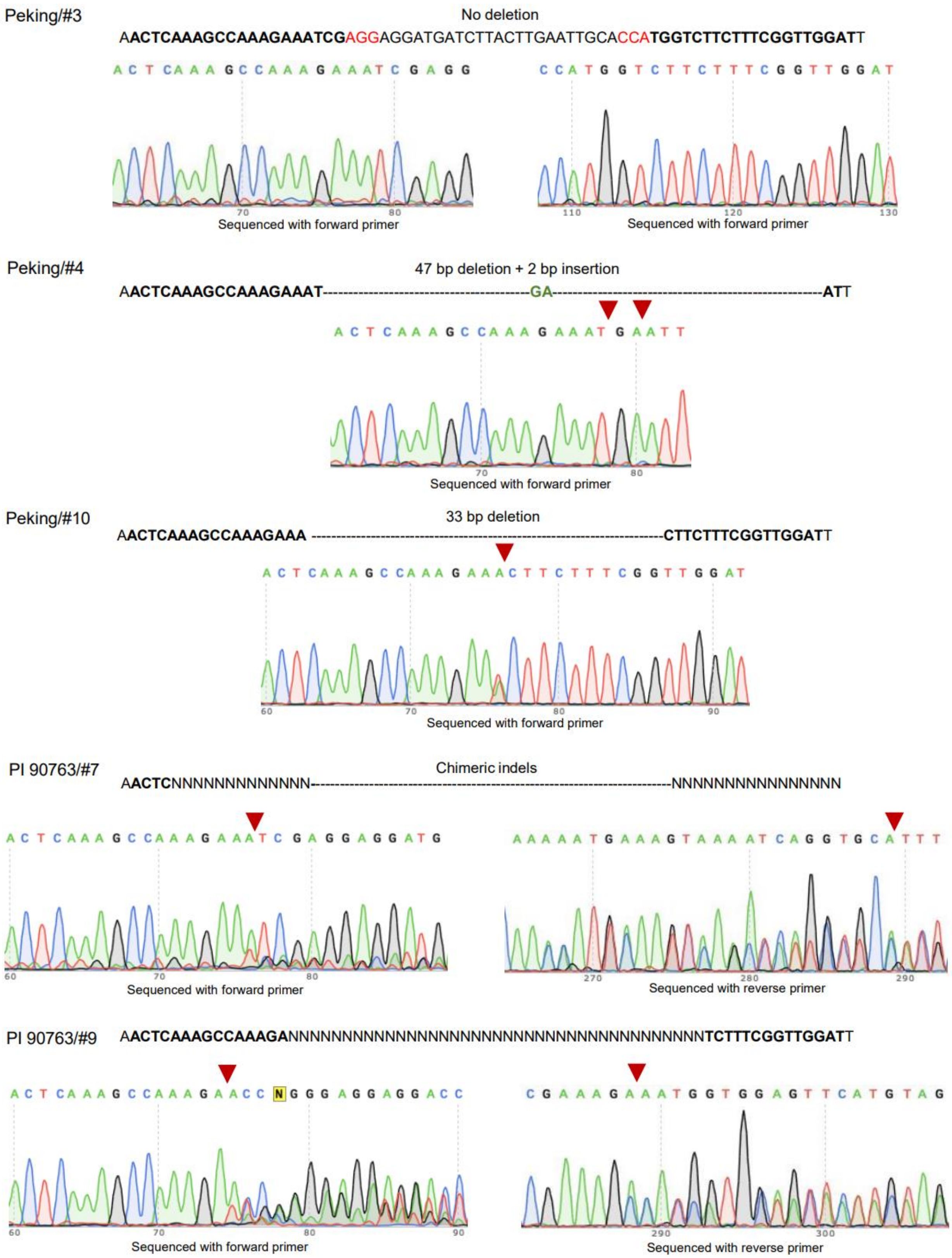

Supplementary Figure 21. Off-target analysis of selected Peking plants transformed with CRISPR/Cas9-*GmSNAP02*-T4+T3. The CHOPCHOP online web tool predicted one off-target sequence for each T4 and T3 gRNA sequence. Both sequences hit *GmSNAP14*, the homolog of *GmSNAP02*. (a) *GmSNAP14*-specific primers were used to amplify the region flanking the two off-target sequences. Genomic DNA was extracted from Peking EV, #1, #5, #6, and #12. The PCR analysis produced a single band with an expected length of 1,149 bp showing no deletions in *GmSNAP14*. (b) Bands from Peking plants #1 and #12 were gel extracted and sequenced. Sequencing confirmed no deletions or insertions at the expected cleavage site. (c) Chromatograms show the absence of deletions at off-target sites in *GmSNAP14*. Red arrows indicate the expected cleavage site.

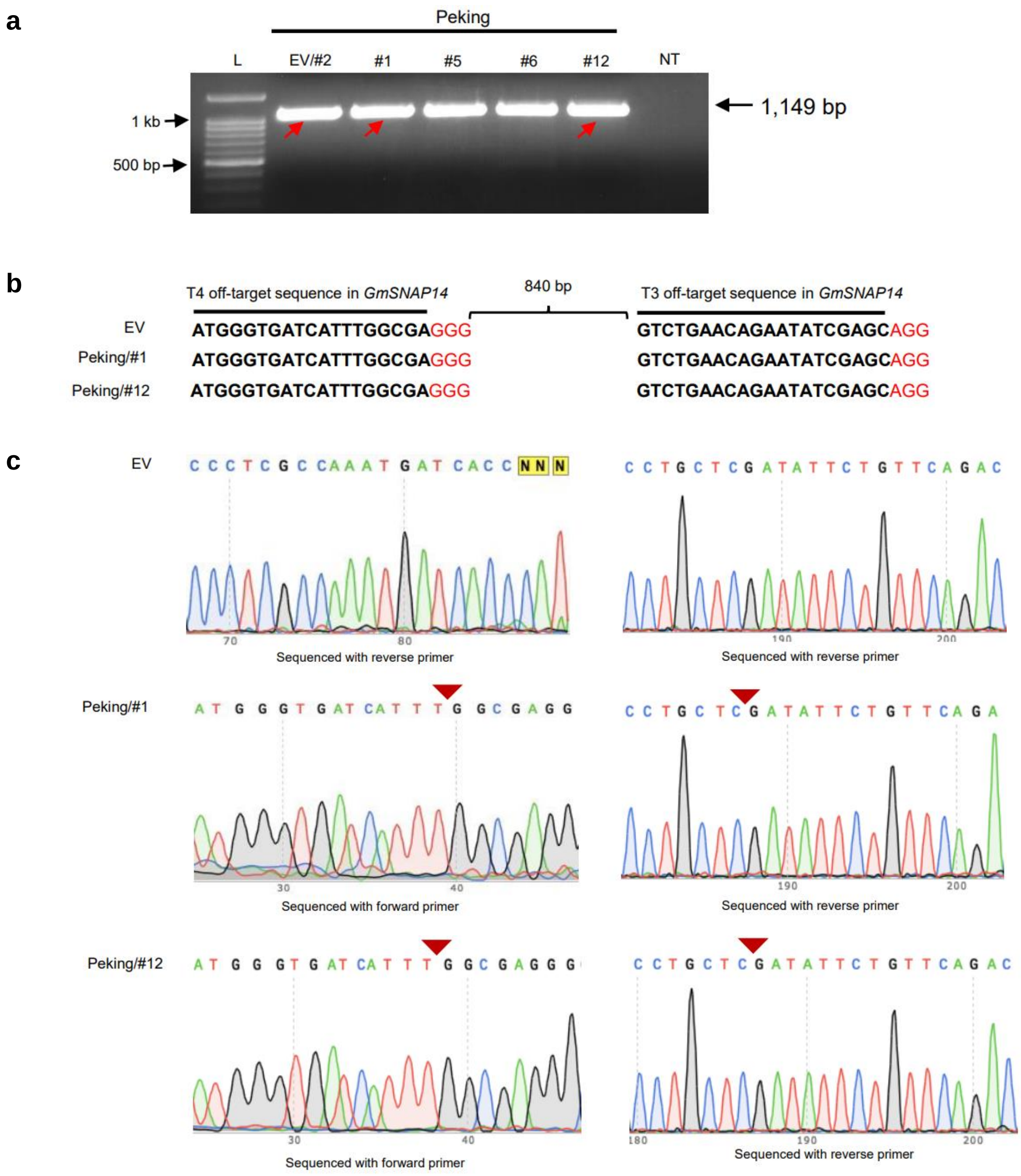

Supplementary Figure 22. Off-target analysis of selected Peking plants transformed with CRISPR/Cas9-*GmSNAP02*-T5+T7. The CHOPCHOP online web tool predicted one off-target sequence for each T5 and T7 gRNA sequence. Only T5 off-target sequences hit *GmSNAP14*, the homolog of *GmSNAP02*. (a) *GmSNAP14*-specific primers were used to amplify the region flanking the off-target sequences. Genomic DNA was extracted from Peking EV, #3, #4, #10, and #13. The PCR analysis produced a single band with an expected length of 413 bp showing no deletions in *GmSNAP14*. (b) Bands from plants #4 and #10 were gel extracted and sequenced. Sequencing confirmed no deletions or insertions at the expected cleavage site. (c) Chromatograms show the absence of deletions at off-target sites in *GmSNAP14*. Red arrows indicate the expected cleavage site.

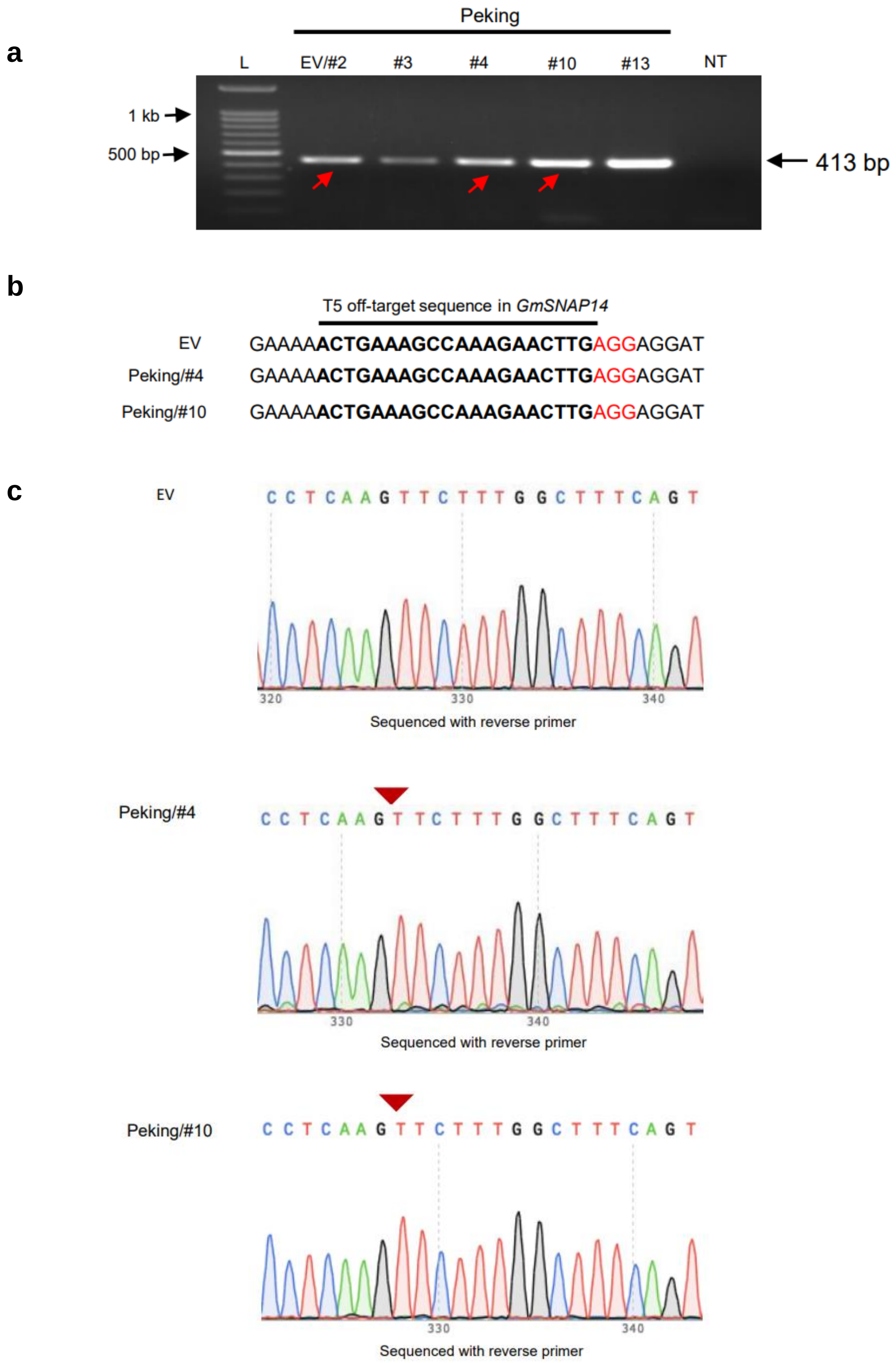

ALLELIC COMBINATIONS SUPPLEMENTARY DATA

Supplementary Figure 23. HG type and race test results at time of testing for four SCN populations: TN7 (HG type 2.5.7; Race 1), TN22 (HG type 1.2.5.7; Race 2), PA3 (HG type 7; Race 3), and MM4 (HG type 2.5.7; Race 5).

		Female Index									
**Population**	**Female # Lee 74**	**Pickett**	**Peking**	**PI 88788**	**PI 90763**	**PI 437654**	**PI 209332**	**PI 89772**	**PI 548316**	**HG Type**	**Race**
**TN7**	235	8	1	90	0	0	99	0	68	2.5.7	1
**TN22**	279	66	16	80	0	0	97	0	70	1.2.5.7	2
**PA3**	379	4	2	4	0	0	8	0	11	7	3
**MM4**	246	16	2	49	0	0	79	0	58	2.5.7	5

ALLELIC COMBINATIONS IN
PI 90763 x Peking

Supplementary Figure 24. Violin plots showing lines with different combinations of homozygous resistance alleles derived from a population (a) PI 90763 x Peking; (b) SA10-8471 x PI 90763; and two populations reported by Basnet et al. 2022: (c) SA13-1385 x PI 90763, and (d) LD11-2170 x PI 90763. The numbers above each plot signify the number of tested lines (n), and letters indicate significant differences between plots based on the Tukey HSD test. The exact P values for each allele combination comparison are provided in the source data file.

a

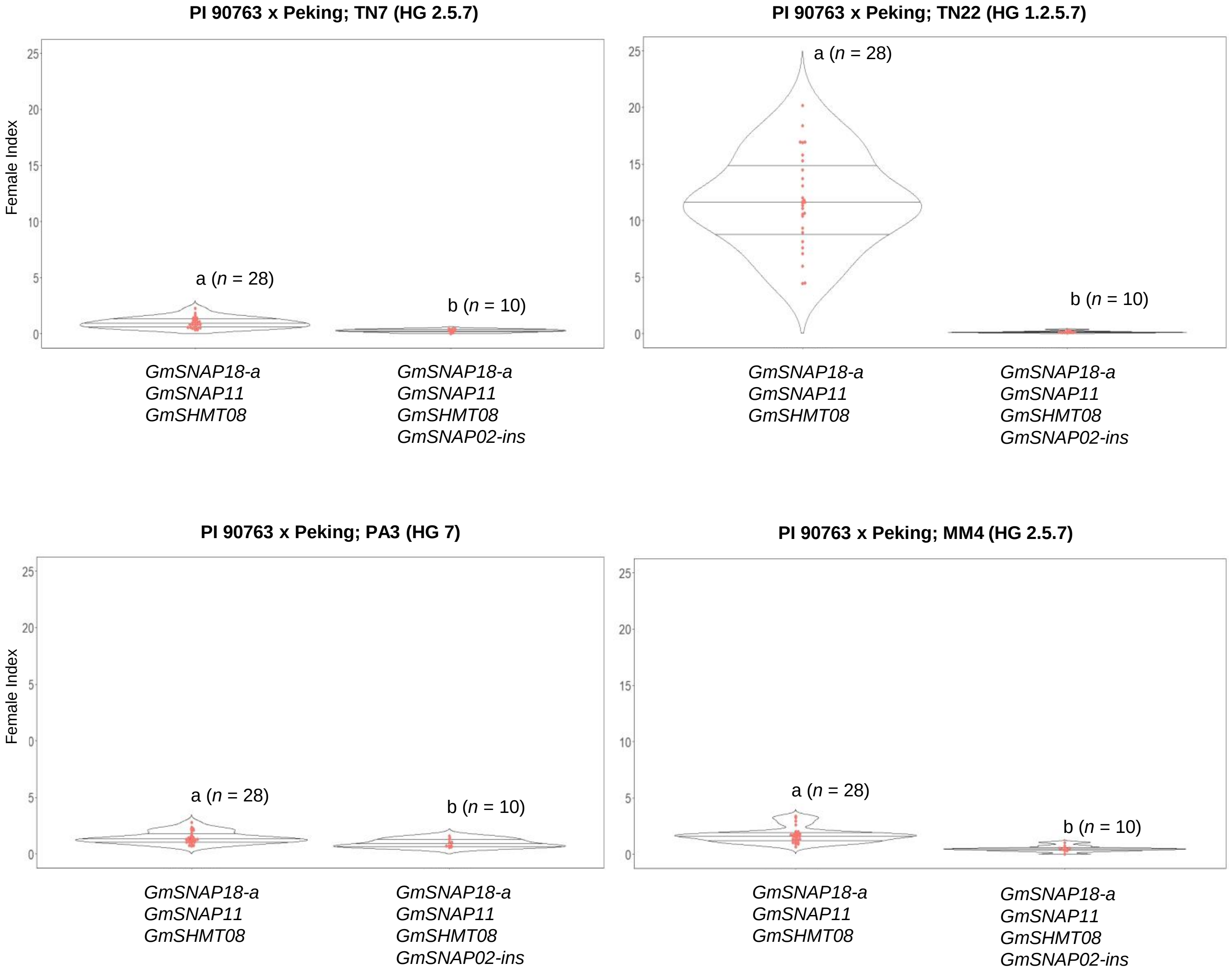

ALLELIC COMBINATIONS IN SA10-8471 x PI 90763

b

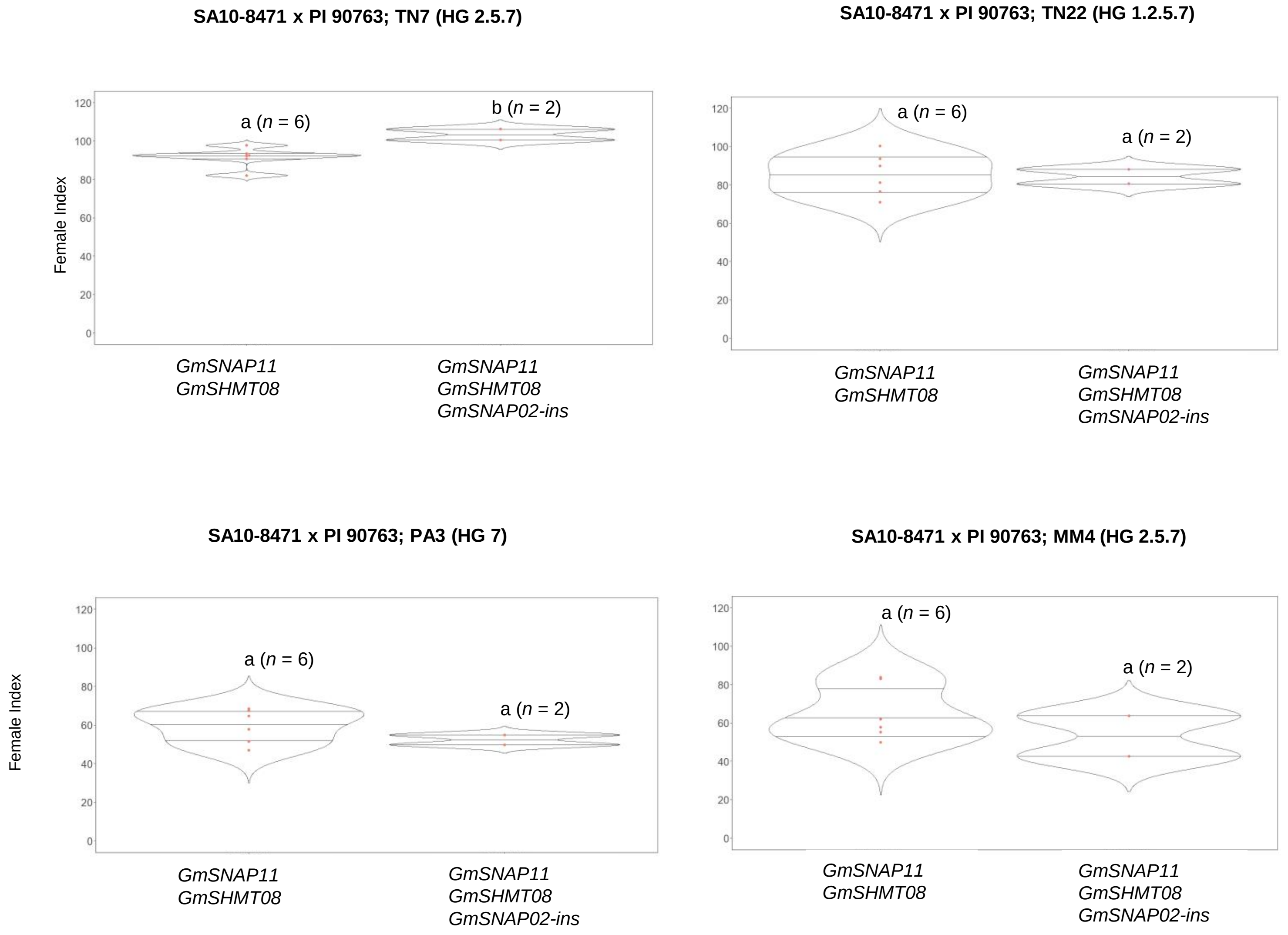

ALLELIC COMBINATIONS IN SA13-1385 x PI 90763

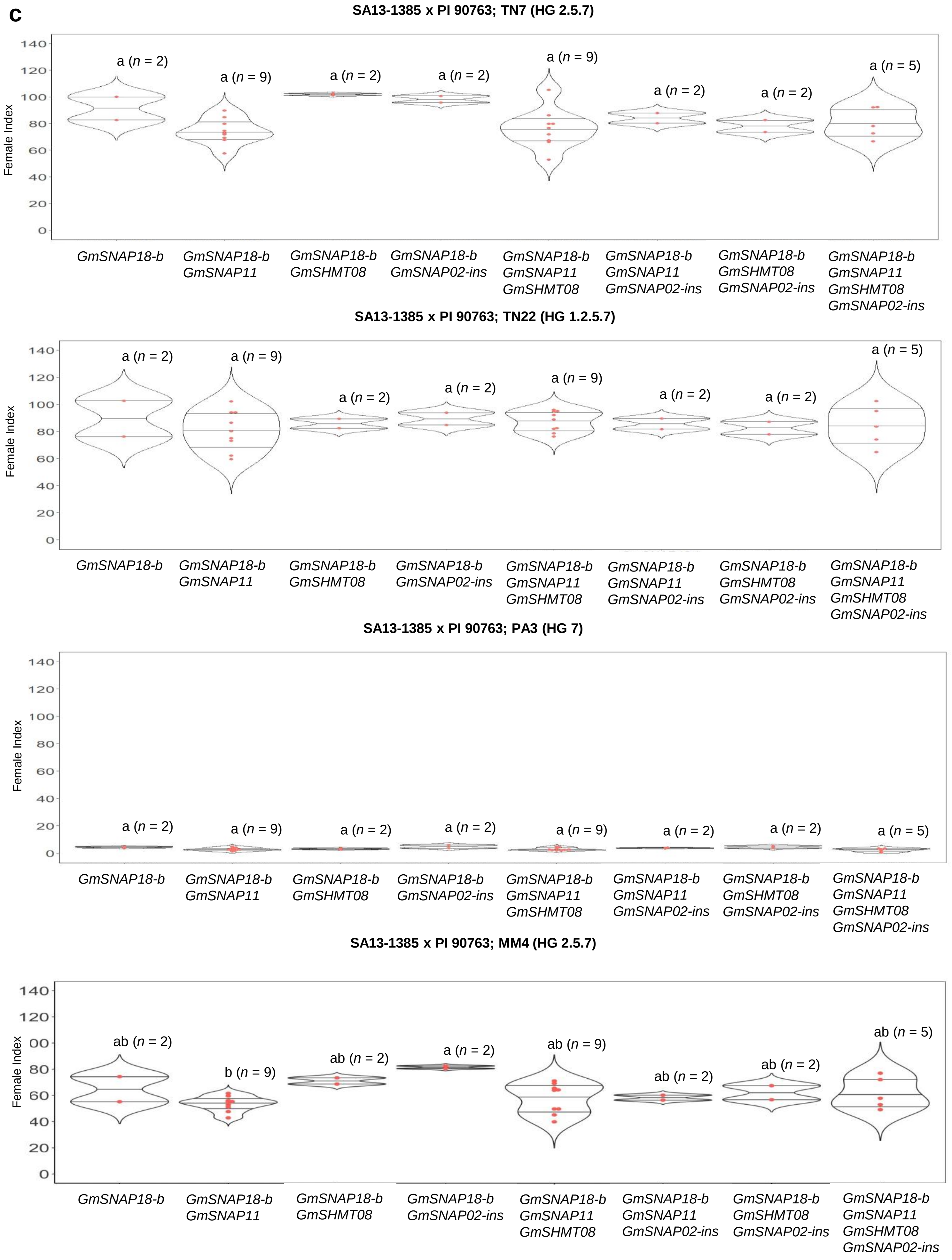

ALLELIC COMBINATIONS IN LD11-2170 x PI 90763

d

LD11-2170 x PI 90763; TN7 (HG 2.5.7)

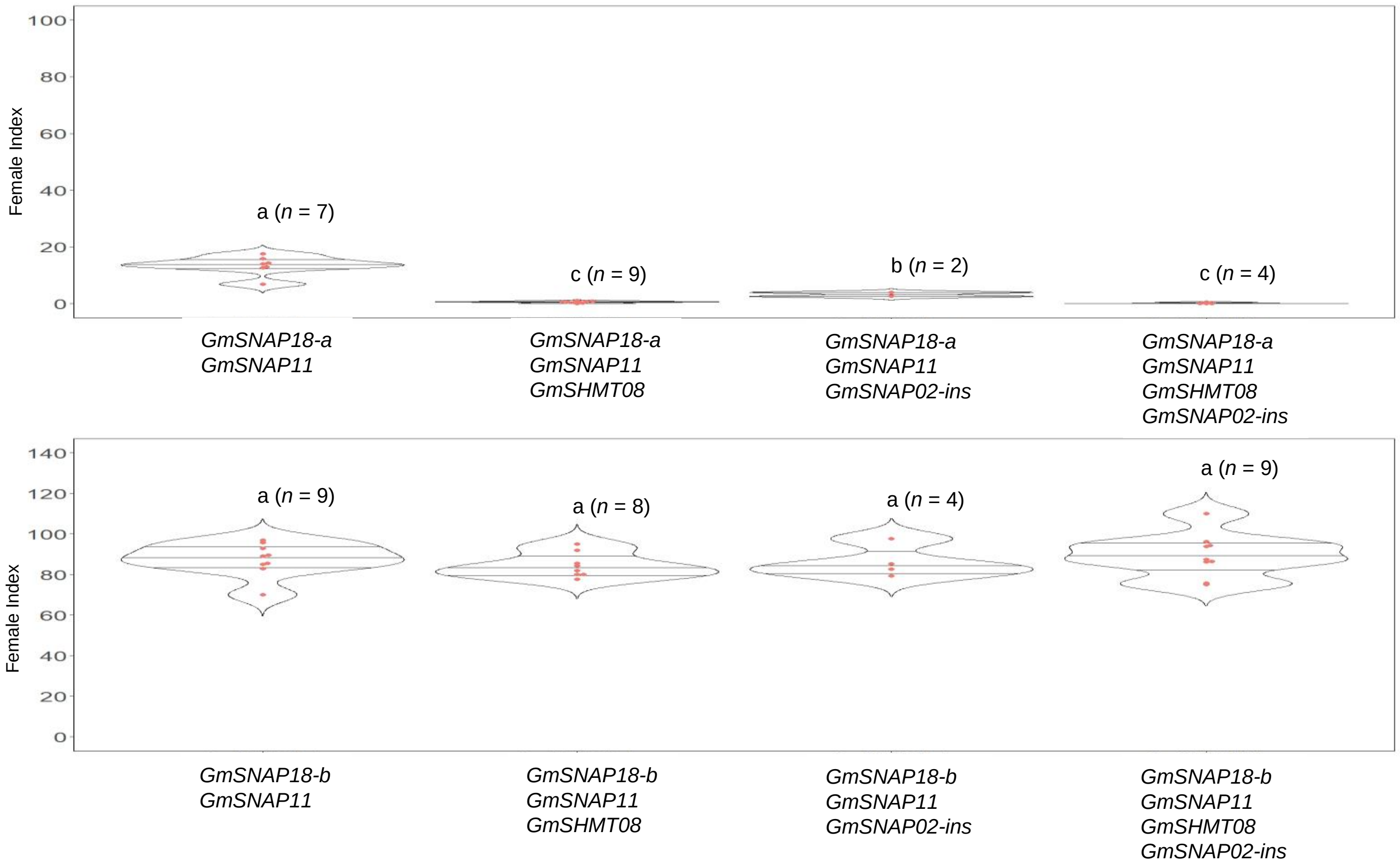

LD11-2170 x PI 90763; TN22 (HG 1.2.5.7)

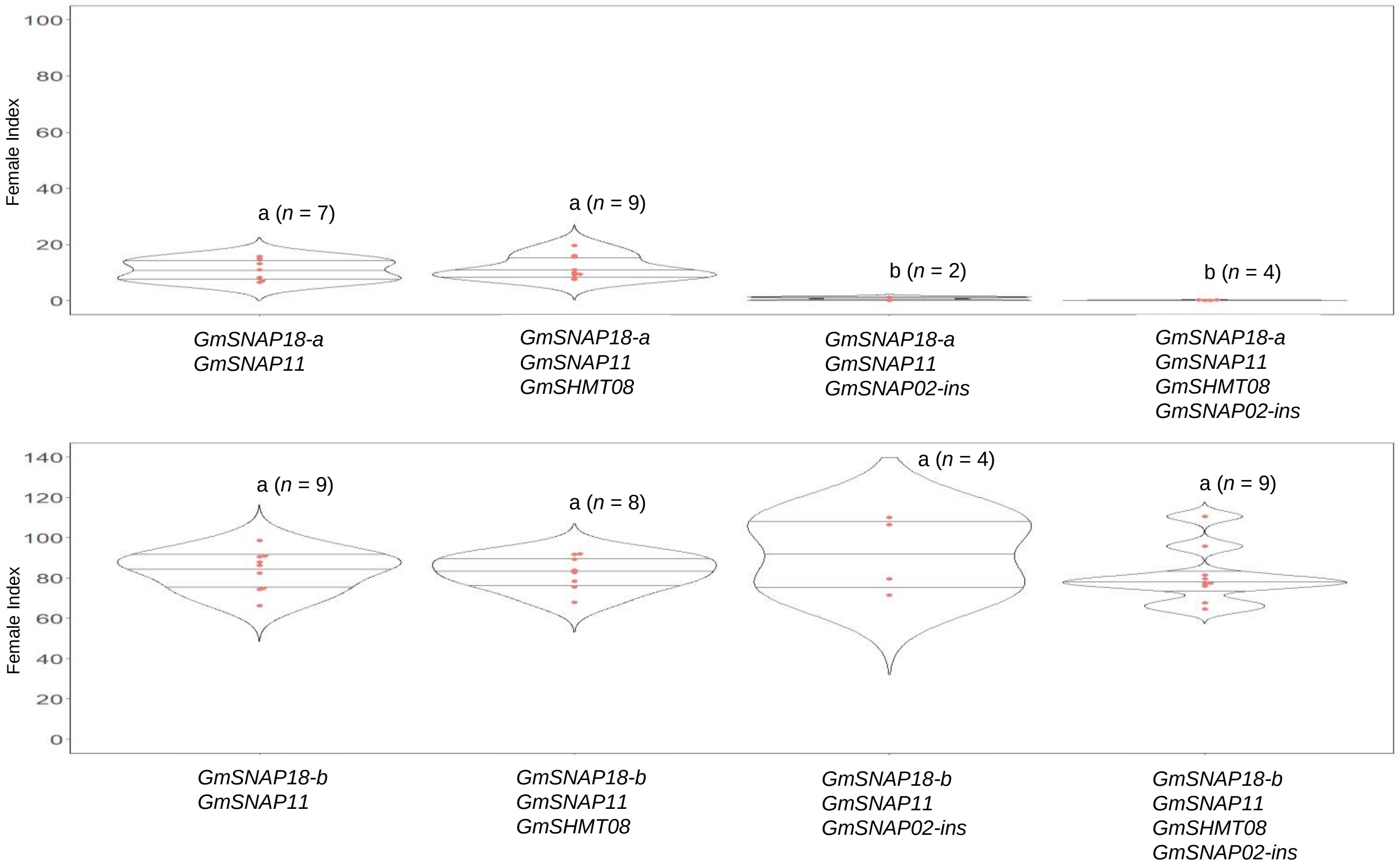

ALLELIC COMBINATIONS IN LD11-2170 x PI 90763

LD11-2170 x PI 90763; PA3 (HG 7)

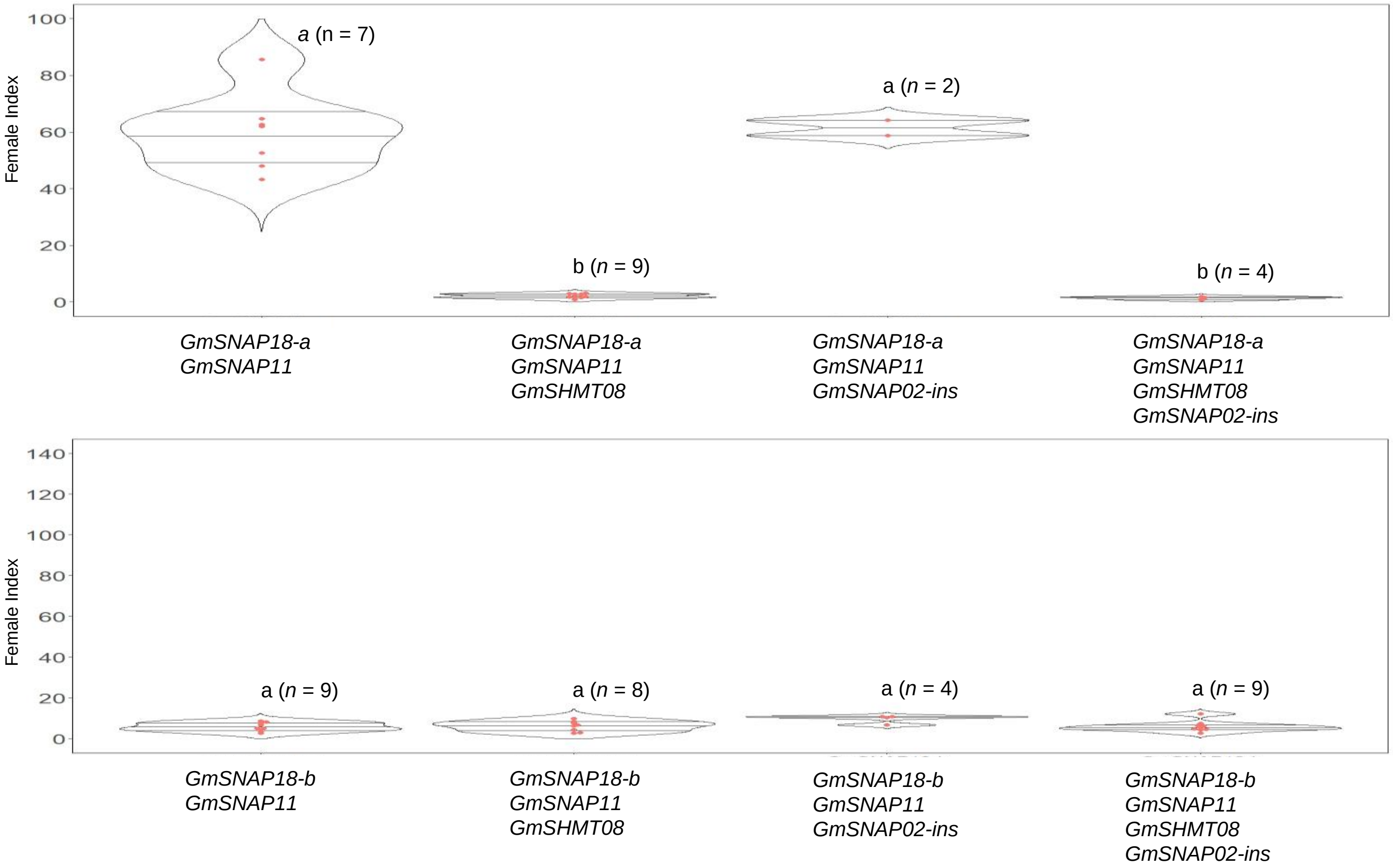

LD11-2170 x PI 90763; MM4 (HG 2.5.7)

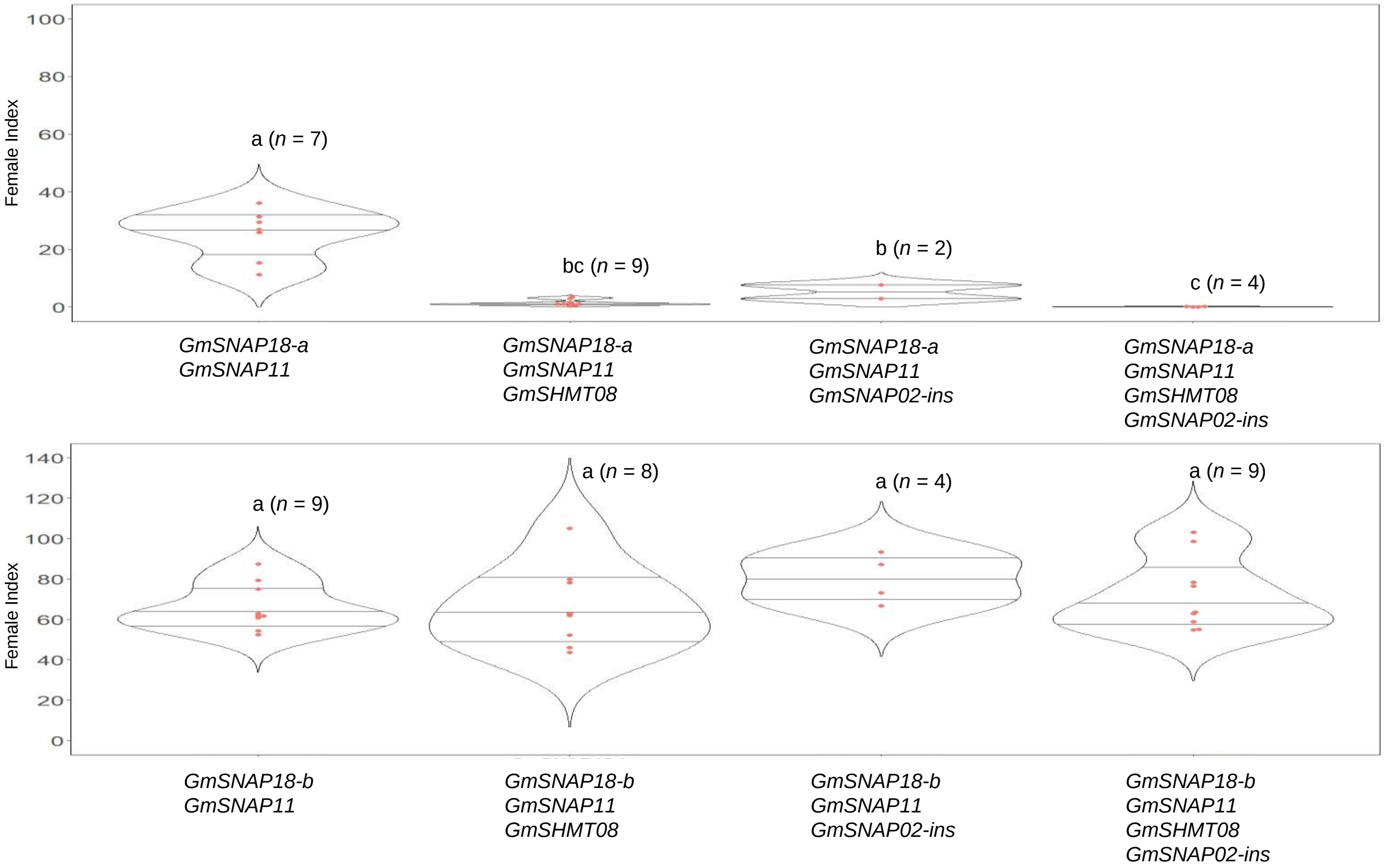

DETECTION OF THE INSERTION IN *GmSNAP02* SUPPLEMENTARY DATA

Supplementary Figure 25. (a) location of a large insertion in exon 8 of *GmSNAP02* resulting in the *GmSNAP02-ins* haplotype. Highlighted in red is unknown sequence of 6 kb; (b) sequences of primers and probes of TaqMan assays MU-SNAP02^{INS}-WT and MU-SNAP02^{INS}-MUT. (c) Endpoint genotyping assays MU-SNAP02^{INS}-WT and MU-SNAP02^{INS}-MUT for a detection of the *GmSNAP02-ins* allele. Scatter plot of fluorescence signals obtained from genomic DNA using SCN resistant checks. Non-amplified samples are indicated in gray.

a

```
Wm82        TCTGGCAGTATGTTTCTAGTTTTGGACATTTCAATAACTGTCTCCTCTGTTATAATGTTC   1920
PI90763     TCTGGCAGTATGTTTCTAGTTTTGGACATTTCAATAACTGTCTCCTCTGTTATAATGTTC   1920
            ************************************************************

Wm82        TTATTAATGTTTGGTTTTATGATAAATTTTATTCTTGCATGTACTGATTCATTGCTCATT   1980
PI90763     TTATTAATGTTTGGTTTTATGATAAATTTTATTCTTGCATGTACTGATTCATTGCTCATT   1980
            ************************************************************

Wm82        TATTTCTGAAGTACTGATGATTTTAAAAAAAATGGGCAGGATATTGCTGCTGCAATTGATG   2040
PI90763     TATTTCTGAAGTACTGATGATTTTAAAAAAAATGGGCAGGATATTGCTGCTGCAATTGATG   2040
            ************************************************************

Wm82        AGGAAGATGTTGGAAAGTTTACTGAAGT--------------------------------   2068
PI90763     AGGAAGATGTTGGAAAGTTTACTGAAGTCACTACTAGAAAAAAGGCTTTTTACGACGGTT   2100
            ****************************

Wm82        ------2--------------------------------------------------TAT   2071
PI90763     AATAAG2CATACAACCGATGTAGATAGGGTGTCGTAAAAAGCTATTTTTGTAGTAGTGTAT   2160
                                                                     ***

Wm82        CAAGGAATTTGATAGTTTGACTCCTTTGGTAAGCTTCAAATTGTTGTAAAATGAAACAGT   2131
PI90763     CAAGGAATTTGATAGTTTGACTCCTTTGGTAAGCTTCAAATTGTTGTAAAATGAAACAGT   2220
            ************************************************************

Wm82        TTTTAGTTGACGCAATGGAGTAAGACTTTATTGTTCTTGATATATAACAAGACTTTTTTC   2191
PI90763     TTTTAGTTGACGCAATGGAGTAAGACTTTATTGTTCTTGATATATAACAAGACTTTTTTC   2280
            ************************************************************

Wm82        AATTTTATTTTTTTGCATACCATAAATAGCTTTGGTCGCGGTAATTAATCAACATTTCAT   2251
PI90763     AATTTTATTTTTTTGCATACCATAAATAGCTTTGGTCGCGGTAATTAATCAACATTTCAT   2340
            ************************************************************
```

b

ASSAY	Wm82	PEKING	PI 90763	HET	Primer-Forward	Primer-Reverse	Probe 1 - VIC-MGB Dye	Probe 2 - FAM-MGB Dye
MU-SNAP02^{INS}-WT	YES	YES	NO	YES	TGATGAGGAAGATGTTGGAAAGT	TCTTACTCCATTGCGTCAACTAA	AGTTTGACTCCTTTGGTAAGCTTCA	AGTTTGACTCCTTTGGTAAGCTTCA
MU-SNAP02^{INS}-MUT	NO	NO	YES	YES	CCGATGTAGATAGGGTGTCGTA	GTCTTACTCCATTGCGTCAACTA	AGTTTGACTCCTTTGGTAAGCTTCA	AGTTTGACTCCTTTGGTAAGCTTCA

MU-SNAP02^{INS}-MUT TaqMan assay detects *GmSNAP02-ins* allele in PI 90763. YES/NO relates to presence/absence of amplification.

c

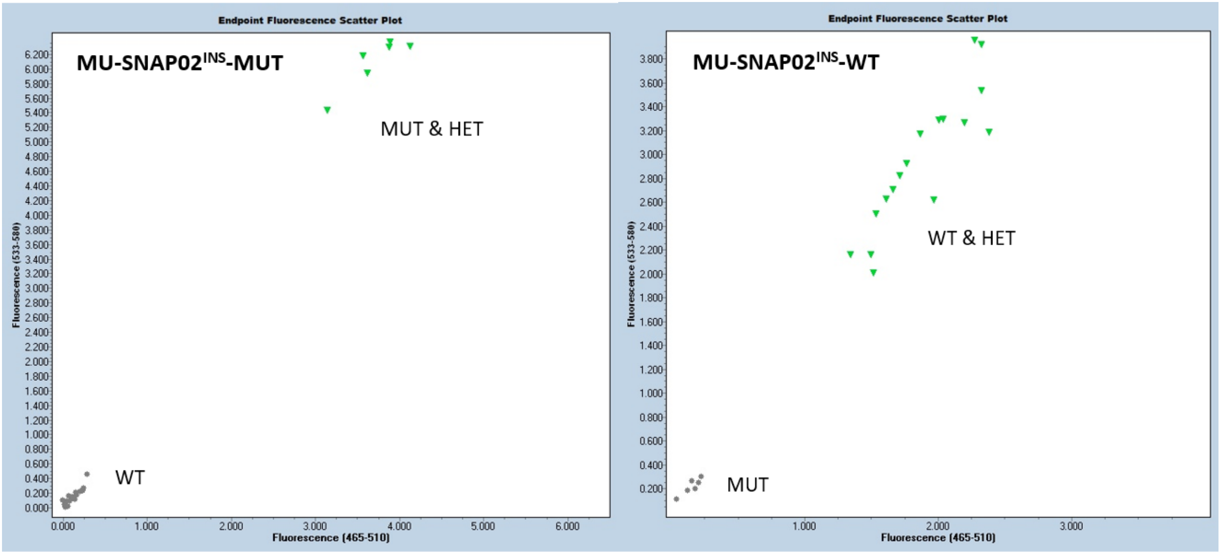

Supplementary Figure 26. (a) Sequences of primers and probes of TaqMan assays MU-SNAP02^{DEL}-1 and MU-SNAP02^{DEL}-2. (b) Endpoint genotyping assays MU-SNAP02^{DEL}-1 and MU-SNAP02^{DEL}-2 for a detection of the *GmSNAP02-del* haplotype. Scatter plot of fluorescence signals obtained from genomic DNA using SCN resistant checks. Non-amplified samples are indicated in gray.

a

ASSAY	Wm82	Forrest	PI 437654	HET	Primer-Forward	Primer-Reverse	Probe 1 - VIC-MGB Dye	Probe 2 - FAM-MGB Dye
MU-SNAP02^{DEL}-1	NEG	Allele X	Allele Y	Both alleles	GATTATTTCATTCATTCCAATATGGGCGAT	CAAGCCCCAACTGCTGAGT	CTGCCTGGCCAAAT	AATCCTCGGCCCTC
MU-SNAP02^{DEL}-2	Allele X	NEG	Allele Y	Both alleles	GATTATTTCATTCATTCCAATATGGGCGAT	CAAGCCCCAACTGCTGAGT	CTGCCTGGCCAAAT	AATCCTCGGCCCTG

MU-SNAP02^{DEL}-1 and MU-SNAP02^{DEL}-2 TaqMan assays detect the *GmSNAP02-del* allele in PI 437654. DEL: no amplification.

b

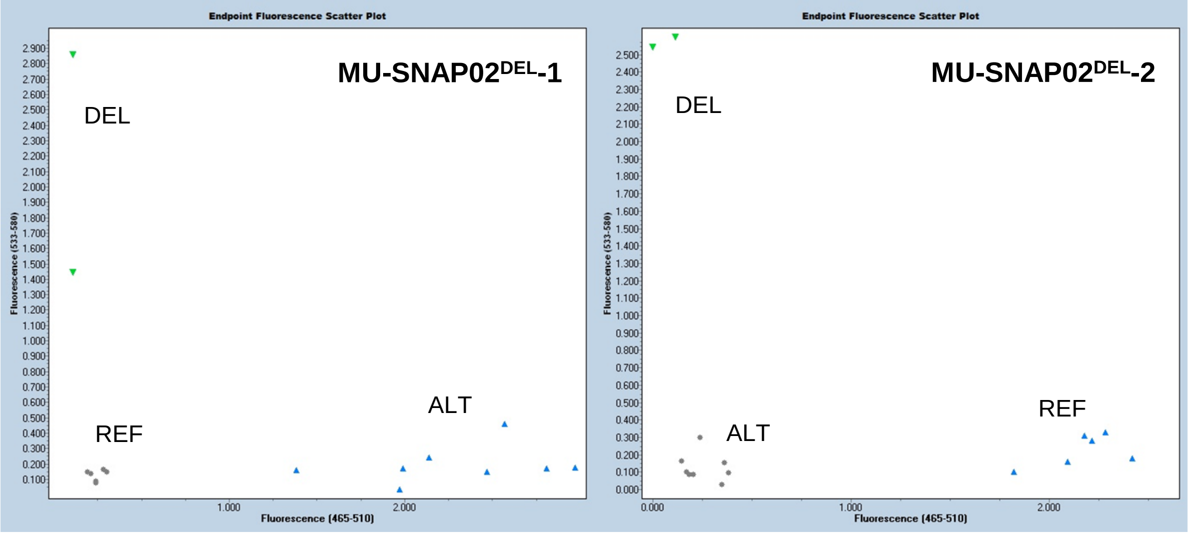

Supplementary Figure 27. Genotypes of selected SCN lines using TaqMan assays for detection of insertion and frameshift of *GmSNAP02*.

	GmSNAP18-b	***GmSNAP18-a***	***GmSNAP11***	***GmSHMT08***	***GmSNAP02-ins***	***GmSNAP02-del***
Williams 82	.	.	.	.	.	.
Lee 74	.	.	.	.	.	.
PI 88788	YES	.	YES	.	.	.
Cloud	YES	.	YES	.	.	.
PI 209332	YES	.	.	.	.	.
Pickett	.	YES	YES	YES	.	.
Peking	.	YES	YES	YES	.	.
PI 567305	.	YES	YES	.	.	.
PI 567516C	.	YES	YES	.	.	.
PI 90763	.	YES	YES	YES	YES	.
PI 507471	YES	.	YES	.	YES	.
PI 603445B	.	YES	YES	YES	YES	.
PI 437654	.	YES	YES	YES	.	YES
PI 89772	.	YES	YES	YES	.	YES
PI 567336A	.	YES	YES	.	.	YES
S05-11482	.	YES	YES	.	.	YES
Hartwig	.	YES	YES	YES	.	YES

GmSNAP18-a and *GmSNAP18-b*: *rhg1-a* and *rhg1-b* alleles detected using Rhg1-2 and SNAP18-1 KASP assays (Kadam et al. 2016; Usovsky et al. 2021).
GmSNAP11: *rhg2* resistant allele detected using SNAP11-1 KASP assay (Usovsky et al. 2021).
GmSHMT08: *Rhg4* resistant allele detected using Rhg4-5 KASP assay (Kadam et al. 2016).
GmSNAP02-ins: resistant allele caused by large insertion in exon 8 of *GmSNAP02* in PI 90763 detected by MU-SNAP02^{INS}-MUT TaqMan assay.
GmSNAP02-del: resistant allele caused by 22-nt deletion in exon 1 of *GmSNAP02* in PI 437654 detected by MU-SNAP02^{DEL}-1 and MU-SNAP02^{DEL}-2 TaqMan assays.
