# Supplementary material for: Loss-of-function of an α-SNAP gene confers resistance to soybean cyst nematode
Source: Nat Commun. 2023 Nov 22;14:7629. doi: 10.1038/s41467-023-43295-y (PMC10665432; doi:10.1038/s41467-023-43295-y)
Supplement: Supplementary file 3 — Reporting Summary [file 41467_2023_43295_MOESM3_ESM.pdf]

nature portfolio

Corresponding author(s): Andrew Scaboo and Melissa Mitchum

Last updated by author(s): 11/03/2023

Reporting Summary

Nature Portfolio wishes to improve the reproducibility of the work that we publish. This form provides structure for consistency and transparency in reporting. For further information on Nature Portfolio policies, see our Editorial Policies and the Editorial Policy Checklist.

Statistics

For all statistical analyses, confirm that the following items are present in the figure legend, table legend, main text, or Methods section.

n/a	Confirmed	
☐	☒	The exact sample size (*n*) for each experimental group/condition, given as a discrete number and unit of measurement
☐	☒	A statement on whether measurements were taken from distinct samples or whether the same sample was measured repeatedly
☐	☒	The statistical test(s) used AND whether they are one- or two-sided<br>*Only common tests should be described solely by name; describe more complex techniques in the Methods section.*
☒	☐	A description of all covariates tested
☐	☒	A description of any assumptions or corrections, such as tests of normality and adjustment for multiple comparisons
☐	☒	A full description of the statistical parameters including central tendency (e.g. means) or other basic estimates (e.g. regression coefficient) AND variation (e.g. standard deviation) or associated estimates of uncertainty (e.g. confidence intervals)
☐	☒	For null hypothesis testing, the test statistic (e.g. *F*, *t*, *r*) with confidence intervals, effect sizes, degrees of freedom and *P* value noted<br>*Give P values as exact values whenever suitable.*
☒	☐	For Bayesian analysis, information on the choice of priors and Markov chain Monte Carlo settings
☒	☐	For hierarchical and complex designs, identification of the appropriate level for tests and full reporting of outcomes
☐	☒	Estimates of effect sizes (e.g. Cohen's *d*, Pearson's *r*), indicating how they were calculated

Our web collection on statistics for biologists contains articles on many of the points above.

Software and code

Policy information about availability of computer code

Data collection	no specific software or code was used to collect data
Data analysis	The following software was used for experiments in this research - RStudio, TASSEL, R packages (ABHgenotypeR, ParentOffSpring), MapQTL 19 6.0, MapChart 2.32, LGC Genomics 1 KASP by design<br><br>Whole genome resequencing data analysis - We analyzed different haplotypes of GmSNAP18, 7 GmSNAP11 and GmSNAP02 using the Soybean Allele Catalog Tool on SoyKB24. Raw sequencing reads 8 of the GmSNAP02 gene (Glyma.02G260400) were obtained from NCBI SRA 9 (https://www.ncbi.nlm.nih.gov/sra) using the fastq-dump command in sra-tools v2.10.0, with the 10 parameters "--gzip --origfmt --split-files". Read quality was assessed using FastQC v 0.11.9 with default 11 parameters and low quality reads and Illumina adapter sequences were trimmed using Trimmomatic v0.39 12 with the parameters "ILLUMINACLIP:TruSeq3-PE.fa:2:30:10:2:True, Leading:3, Trailing:3, 13 MINLEN:36". Quality-trimmed reads were aligned to the Williams 82 reference genome (Wm82.a2.v1 14 from Phytozome - https:// phytozome-next.jgi.doe.gov/) using the bwa mem command in BWA v0.7.17 15 with default parameters. Mate coordinates and size fields were filled using the fixmate command in 16 Samtools v1.13 with parameter "-m" to add mate score tags. Reads were coordinate sorted using samtools 17 sort and duplicate reads marked with samtools markdup. Reads aligned to the region on Chr. 2 containing 18 the GmSNAP02 gene were extracted using samtools view command and visually inspected in the JBrowse 19 2 v1.7.7 desktop genome browser. Variant calling on all accessions were performed using the GATK 20 v4.1.9.0 platform as previously described56.

For manuscripts utilizing custom algorithms or software that are central to the research but not yet described in published literature, software must be made available to editors and reviewers. We strongly encourage code deposition in a community repository (e.g. GitHub). See the Nature Portfolio guidelines for submitting code & software for further information.

Data

Policy information about availability of data

All manuscripts must include a data availability statement. This statement should provide the following information, where applicable:

- Accession codes, unique identifiers, or web links for publicly available datasets
- A description of any restrictions on data availability
- For clinical datasets or third party data, please ensure that the statement adheres to our policy

The authors declare that the data supporting the findings of this study are available within the paper, within the supplementary information file, and within the data source file. The raw whole genome sequencing data used within this paper are available for the four accessions as follows; PI 90763 [https://www.ncbi.nlm.nih.gov/sra/?term=SRR2163296], PI 437654 [https://www.ncbi.nlm.nih.gov/sra/?term=SRR2163307], Peking [https://www.ncbi.nlm.nih.gov/sra/?term=SRR2163294], and Williams 82 [https://ngdc.cncb.ac.cn/gsa/browse/CRA002269/CRR108703]. Plant material and soybean cyst nematode populations used in this manuscript are available upon request via a material transfer agreement and associated permits with each respective institution.

Research involving human participants, their data, or biological material

Policy information about studies with human participants or human data. See also policy information about sex, gender (identity/presentation), and sexual orientation and race, ethnicity and racism.

Reporting on sex and gender	not applicable
Reporting on race, ethnicity, or other socially relevant groupings	not applicable
Population characteristics	not applicable
Recruitment	not applicable
Ethics oversight	not applicable

Note that full information on the approval of the study protocol must also be provided in the manuscript.

Field-specific reporting

Please select the one below that is the best fit for your research. If you are not sure, read the appropriate sections before making your selection.

☒ Life sciences ☐ Behavioural & social sciences ☐ Ecological, evolutionary & environmental sciences

For a reference copy of the document with all sections, see nature.com/documents/nr-reporting-summary-flat.pdf

Life sciences study design

All studies must disclose on these points even when the disclosure is negative.

Sample size	Sample sizes for experiments were designed via standard and relevant scientific protocol for all experimental procedures used
Data exclusions	No data were excluded
Replication	All experiments employed replication, including biological and experimental replications
Randomization	Randomization was employed for all experiments that called for such a design including bio-assays
Blinding	not applicable

Behavioural & social sciences study design

All studies must disclose on these points even when the disclosure is negative.

Study description	not applicable
Research sample	not applicable
Sampling strategy	not applicable
Data collection	not applicable

Timing	not applicable
Data exclusions	not applicable
Non-participation	not applicable
Randomization	not applicable

Ecological, evolutionary & environmental sciences study design

All studies must disclose on these points even when the disclosure is negative.

Study description	not applicable
Research sample	not applicable
Sampling strategy	not applicable
Data collection	not applicable
Timing and spatial scale	not applicable
Data exclusions	not applicable
Reproducibility	not applicable
Randomization	not applicable
Blinding	not applicable

Did the study involve field work? ☐ Yes ☒ No

Field work, collection and transport

Field conditions	not applicable
Location	not applicable
Access & import/export	not applicable
Disturbance	not applicable

Reporting for specific materials, systems and methods

We require information from authors about some types of materials, experimental systems and methods used in many studies. Here, indicate whether each material, system or method listed is relevant to your study. If you are not sure if a list item applies to your research, read the appropriate section before selecting a response.

Materials & experimental systems

n/a	Involved in the study	
☒	☐	Antibodies
☒	☐	Eukaryotic cell lines
☒	☐	Palaeontology and archaeology
☐	☒	Animals and other organisms
☒	☐	Clinical data
☒	☐	Dual use research of concern
☐	☒	Plants

Methods

n/a	Involved in the study	
☒	☐	ChIP-seq
☒	☐	Flow cytometry
☒	☐	MRI-based neuroimaging

Antibodies

Antibodies used	not applicable

Validation | not applicable

Eukaryotic cell lines

Policy information about cell lines and Sex and Gender in Research

Cell line source(s)	not applicable
Authentication	not applicable
Mycoplasma contamination	not applicable
Commonly misidentified lines (See ICLAC register)	not applicable

Palaeontology and Archaeology

Specimen provenance	not applicable
Specimen deposition	not applicable
Dating methods	not applicable

☐ Tick this box to confirm that the raw and calibrated dates are available in the paper or in Supplementary Information.

Ethics oversight	not applicable

Note that full information on the approval of the study protocol must also be provided in the manuscript.

Animals and other research organisms

Policy information about studies involving animals; ARRIVE guidelines recommended for reporting animal research, and Sex and Gender in Research

Laboratory animals	Heterodera glycines Inbred Populations TN7 (HG type 2.5.7; Race 1), TN22 (HG type 1.2.5.7; Race 2), PA3 (HG type 7; Race 3), MM5 (HG type 2.5.7; Race 5)
Wild animals	not applicable
Reporting on sex	not applicable
Field-collected samples	not applicable
Ethics oversight	not applicable

Note that full information on the approval of the study protocol must also be provided in the manuscript.

Clinical data

Policy information about clinical studies
All manuscripts should comply with the ICMJEguidelines for publication of clinical research and a completedCONSORT checklist must be included with all submissions.

Clinical trial registration	not applicable
Study protocol	not applicable
Data collection	not applicable
Outcomes	not applicable

Dual use research of concern

Policy information about dual use research of concern

Hazards

Could the accidental, deliberate or reckless misuse of agents or technologies generated in the work, or the application of information presented in the manuscript, pose a threat to:

No	Yes	
☒	☐	Public health
☒	☐	National security
☒	☐	Crops and/or livestock
☒	☐	Ecosystems
☒	☐	Any other significant area

Experiments of concern

Does the work involve any of these experiments of concern:

No	Yes	
☒	☐	Demonstrate how to render a vaccine ineffective
☒	☐	Confer resistance to therapeutically useful antibiotics or antiviral agents
☒	☐	Enhance the virulence of a pathogen or render a nonpathogen virulent
☒	☐	Increase transmissibility of a pathogen
☒	☐	Alter the host range of a pathogen
☒	☐	Enable evasion of diagnostic/detection modalities
☒	☐	Enable the weaponization of a biological agent or toxin
☒	☐	Any other potentially harmful combination of experiments and agents

Plants

Seed stocks

Seed stocks of plant germplasm used in this study are available upon request from the United States Department of Agriculture and/or the University of Missouri

Novel plant genotypes

GmSNAP02 CRISPR guide RNA (gRNA) and construct design - A dual sgRNA plasmid construction system was used as described in Kang, 201662. The CRISPR/Cas9 construct backbone 35S-Cas9-SK and sgRNA template plasmid AtU6-26-SK were gifts from Jiam-Kang Zhu's lab. This system utilizes the Arabidopsis AtU6-26 promotor to drive expression of the human codon-optimized Streptococcus pyogenes Cas9 (hspCas9)25,63. Several GmSNAP02 gRNA sequences for CRISPR/Cas9 targeting different regions of GmSNAP02 were designed using the CHOPCHOP web tool64. Complementary oligomers for selected gRNA were designed and annealed double-stranded gRNA sequences were cloned into the AtU6-26-SK vector. The Cas9 cassette from 35S-Cas9-SK and the sgRNA expression cassettes were subcloned into the pcamGFP-CvMV-GWOX binary vector. The pcamGFP-CvMV-GWOX binary vector has a strong CvMV promoter driving a GFP reporter gene cassette for transgene selection. The final constructs were introduced into the Rhizobium rhizogenes strain K599 using the freeze-thaw method65. Two different pcamGFP-CvMV-GmSNAP02-gRNAa-gRNAb constructs confirmed for GmSNAP02 edits by sequencing were used to generate soybean composite plants with transgenic roots. The final constructs were named as pcamGFP-CvMV-GmSNAP02-T3-T4 and pcamGFP-CvMV-GmSNAP02-T5-T7. The pcamGFP-CvMV-GWOX construct with no gRNA was used as the empty vector (EV) control. Primers used for vector construction are in the Supplementary File S12.

Generation of composite soybean plants - Composite soybean plants with GmSNAP02-edited roots were generated by Rhizobium rhizogenes (K599) mediated transformation as previously described66 with modifications for SCN bioassays. Following K599 inoculation, plants were sealed and kept in a growth chamber set to 26°C with a photoperiod of 16h light/8h dark for hairy root generation before moving to the greenhouse. Composite plants with transformed hairy roots were selected under fluorescent light and untransformed roots were removed. Plants with uniform roots were transplanted to 1:1 sand:soil mix for SCN bioassays. Two independent biological replicates, each containing 14 plants for two different GmSNAP02 knock-out events and EV were used for phenotyping. The first and second replicates of soybean composite plants were inoculated with 3,000 and 2,000 SCN HG type 1.2.5.7 eggs, respectively. Inoculated plants were kept in the greenhouse at 26°C with a 16h/8h light/dark period for 28 days. Cysts from roots were extracted and counted using a stereoscope. A Kruskal–Wallis statistical test was used to determine significant differences in cyst counts in between Peking plants transformed with different CRISPR/Cas9-gRNAa+gRNAb constructs. Since there was a significant difference, we carried out a pair-wise comparison using the Wilcoxon rank sums test. The dot plot was created using the R program ver. 4.2.2.

Plant populations development - Three recombinant inbred populations composed of 144, 131, and 246 F3:4 lines were developed from crosses between PI 90763 × Peking, Forrest × PI 437654, and SA10-8471 × PI 90763, respectively, and population development was similar to the populations described previously12. All cross hybridizations were made at the Bay Farm Research Facility in Columbia, Missouri during the summer of 2019 and populations were advanced using single-seed descent at winter nurseries in Hawaii and/or Puerto Rico before composite line establishment in later filial generations.

Authentication

Endpoint genotyping with KASP and Taqman markers was used to validate true hybridizations from cross-pollination and during subsequent population development

ChIP-seq

Data deposition

☐ Confirm that both raw and final processed data have been deposited in a public database such as GEO.

☐ Confirm that you have deposited or provided access to graph files (e.g. BED files) for the called peaks.

Data access links<br>*May remain private before publication.*	not applicable
Files in database submission	not applicable
Genome browser session<br>(e.g. UCSC)	not applicable

Methodology

Replicates	not applicable
Sequencing depth	not applicable
Antibodies	not applicable
Peak calling parameters	not applicable
Data quality	not applicable
Software	not applicable

Flow Cytometry

Plots

Confirm that:

☐ The axis labels state the marker and fluorochrome used (e.g. CD4-FITC).

☐ The axis scales are clearly visible. Include numbers along axes only for bottom left plot of group (a 'group' is an analysis of identical markers).

☐ All plots are contour plots with outliers or pseudocolor plots.

☐ A numerical value for number of cells or percentage (with statistics) is provided.

Methodology

Sample preparation	not applicable
Instrument	not applicable
Software	not applicable
Cell population abundance	not applicable
Gating strategy	not applicable

☐ Tick this box to confirm that a figure exemplifying the gating strategy is provided in the Supplementary Information.

Magnetic resonance imaging

Experimental design

Design type	not applicable
Design specifications	not applicable
Behavioral performance measures	not applicable

Acquisition

Imaging type(s)	not applicable
Field strength	not applicable
Sequence & imaging parameters	not applicable
Area of acquisition	not applicable

Diffusion MRI ☐ Used ☐ Not used

Preprocessing

Preprocessing software	not applicable
Normalization	not applicable
Normalization template	not applicable
Noise and artifact removal	not applicable
Volume censoring	not applicable

Statistical modeling & inference

Model type and settings	not applicable
Effect(s) tested	not applicable

Specify type of analysis: ☐ Whole brain ☐ ROI-based ☐ Both

Statistic type for inference (See Eklund et al. 2016)	not applicable
Correction	not applicable

Models & analysis

n/a	Involved in the study
☐	☐ Functional and/or effective connectivity
☐	☐ Graph analysis
☐	☐ Multivariate modeling or predictive analysis

Functional and/or effective connectivity	not applicable
Graph analysis	not applicable
Multivariate modeling and predictive analysis	not applicable
